# Cellular microenvironment of erythropoietin‐producing cells in hypoxic and injured mouse kidneys

**DOI:** 10.1113/EP093422

**Published:** 2026-01-08

**Authors:** Olga M. Lempke, Thomas Knöpfel, Stellor Nlandu Khodo, Roland H. Wenger

**Affiliations:** ^1^ Institute of Physiology University of Zürich Zürich Switzerland

**Keywords:** erythropoietin, hypoxia‐inducible factor, PHD inhibitor, roxadustat, sodium glucose transporter, spatial transcriptomics

## Abstract

The main sources of circulating erythropoietin (Epo) in the adult are kidney Norn cells, a recently identified interstitial cell type capable of becoming renal Epo‐producing (REP) cells following a local decrease in tissue oxygenation. REP cells are restricted to small clusters in the corticomedullary border region, suggesting that their microenvironment is relevant for cell differentiation and/or proper regulation of Epo production. Possibly for the same reason, REP cells cease to produce Epo in injured kidneys, which is rapidly reverted by stabilizers of the hypoxia‐inducible factor (HIF). To shed new light on the mechanisms governing Epo production, we combined spatial transcriptomics, mRNA fluorescence in situ hybridization and sequential immunofluorescence, enabling the characterization of the immediate neighbourhood of active REP cells. Although in the hypoxic mouse kidney REP cells were closest to proximal tubule segments (S) S1 to S2/3 and endothelial cells, Epo was reinduced by HIF stabilizers in injured kidneys in the vicinity of damaged proximal tubule cells that expressed high levels of injury markers. In contrast, the Norn and endothelial cell profiles remained normal. The REP cell microenvironment switched from pathways involved in energy metabolism in hypoxic conditions to inflammatory and fibrotic pathways in injury conditions. In summary, these data demonstrate that in the diseased kidney HIF stabilizers reinduce Epo expression in REP cells with a metabolically inactive proximal tubule neighbourhood, consistent with a causal role for tubular cells during the loss of Epo expression.

## Abstract (highlights)

- **What is the central question of this study?**
  Renal erythropoietin (Epo)‐producing (REP) cells are non‐randomly distributed, implying that the local milieu of these cells is relevant for hypoxia‐inducible Epo expression and for the loss of Epo production in end‐stage renal disease.
- **What is the main finding and its importance?**
  By using spatial transcriptomics, mRNA fluorescence in situ hybridization and sequential immunofluorescence, we demonstrate that REP cells remain intact even in severely injured mouse kidneys, suggesting that the loss of Epo is caused mainly by the observed tissue damage and metabolic changes of cells in the immediate neighbourhood rather than by REP cells themselves.

## 1 INTRODUCTION

The endocrine hormone erythropoietin (Epo) is essential for regulating erythropoiesis in the red bone marrow (Wenger & Kurtz, 2011). In adults, Epo is produced mainly by the kidneys. Consequently, anaemic patients suffering from end‐stage renal disease commonly require erythropoiesis‐stimulating agents (ESAs), such as recombinant Epo or stabilizers of the hypoxia‐inducible factor (HIF), the main transcriptional regulator of Epo production (Haase, 2021). In conditions of systemic hypoxia, attributable to oxygen deprivation or anaemia, HIF‐2 activation ensures adequate Epo production to normalize oxygen supply to tissues. The kidney cells actively expressing the *Epo* gene, herein after referred to as renal Epo‐producing (REP) cells, are peritubular interstitial cells whose origin and identity remained enigmatic for decades (Dahl et al., 2022a). Therefore, we previously developed a transgenic mouse model to label REP cells permanently (Imeri et al., 2019). These mice allowed for the isolation and enrichment of REP cells and, combined with modern scRNAseq and scATACseq techniques, resulted in the identification of a new renal cell type named Norn cells (Kragesteen et al., 2023). Norn cells share fibroblastic, pericytic and neuronal features, and encompass all renal interstitial cells with the potential to produce Epo, even if at any given time point only a subfraction of Norn cells are active REP cells (Bapst et al., 2022; Dahl et al., 2022b; Imeri et al., 2019; Kragesteen et al., 2023).

REP cells are not randomly distributed in the kidney but appear as small clusters in the corticomedullary border region. Despite the relatively hypoxic tissue microenvironment imposed by the tubulovascular topography, REP cells are detected only sporadically, if at all, in the inner medulla, suggesting that their precise location is relevant for the maintenance of cell differentiation and/or the proper regulation of *Epo* gene expression (Dahl et al., 2022a; Nolan & Wenger, 2018). Furthermore, it is still poorly understood why Norn cells lose their ability to become REP cells during chronic kidney disease (CKD) progression, resulting in renal anaemia. According to one hypothesis, Epo production in REP cells is suppressed as a consequence of myofibroblast transdifferentiation (Souma et al., 2013; Suzuki & Yamamoto, 2016). However, although we found a strong increase in myofibroblasts during kidney damage, labelled REP cells lost Epo production without an increase in myofibroblast markers (Dahl et al., 2022b). Moreover, the myofibroblast transdifferentiation hypothesis cannot explain why HIF‐stabilizing agents rapidly reinduce Epo mRNA production in these damaged kidneys (Dahl et al., 2022b). Another assumption potentially explaining Epo reinduction is the presence of microregions in the otherwise damaged kidney, which are still sufficiently functional to compensate for the loss of Epo production after treatment with HIF‐stabilizing agents (Kobayashi et al., 2022).

To shed new light on these open questions, we used spatial transcriptomics, mRNA fluorescence in situ hybridization (mRNA‐FISH) and sequential immunofluorescence to investigate the cells neighbouring REP cells in hypoxic kidneys and kidneys following unilateral ureteral obstruction (UUO) and treatment with the HIF stabilizer FG‐4592/roxadustat. Special attention was given to the vicinity of REP cells, notably, to the proximal tubule (PT) segments S1 and S2/S3, positive for sodium–glucose cotransporter (Sglt) Sglt2 (*Slc5a2*) and Sglt1 (*Slc5a1*), respectively (Limbutara et al., 2020). Because sodium transport in the PT is associated with high oxygen consumption, the precise spatial relationship between REP cells and PT S1–S2/S3 tubular epithelia is likely to be of major relevance for the microenvironmental tissue $P_{O_{2}}$ dictating *Epo* gene expression.

## 2 MATERIALS AND METHODS

### 2.1 Ethical approval

The mouse studies conformed to the standards set by the Swiss Animal Welfare Act and are in compliance with the institutional guidelines and the policies of *Experimental Physiology* for animal research. All animal experiments had been approved by the cantonal commission for animal experimentation and the veterinary office of the canton Zurich, Switzerland (licence numbers ZH233/2015, ZH200/2016 and ZH085/2019). All procedures were designed and conducted to minimize pain and distress, and animals were closely monitored throughout the study to ensure their welfare.

### 2.2 Mice

The tissues reused for this study were selected based on RNA quality control and were part of a larger animal investigation that has been reported in detail previously (Dahl et al., 2022b; Imeri et al., 2019). Briefly, mice included in the present study were homozygous for the *Epo‐CreERT2#1* [B6D2;C57BL6N‐Tg(EPO::Cre)nRhw] and Ai14 reporter [B6.Cg‐Gt(ROSA)26Sor<tm14(CAG‐tdTomato)Hze>/J] alleles and contained the corresponding genetic backgrounds (Imeri et al., 2019). A total of 21 mice were included, aged 4–8 weeks and weighing 20–30 g (male) and 18–25 g (female). The sex and number of mice for each analysis are provided in the corresponding figure legends. Sex‐independent Epo expression has previously been demonstrated in these mice (Dahl et al., 2022b).

Mice were maintained on a 12 h–12 h light–dark cycle in standard housing conditions with two to five animals per cage, enriched with nesting material and shelters, at 20°C–24°C and 40%–60% humidity, and with ad libitum access to food and water. Animal care, housing and experimentation were performed by the Laboratory Animal Services Center (LASC) and the Zurich Integrative Rodent Physiology (ZIRP) facilities of the University of Zurich. Mice were identified by ear notching in accordance with LASC licence 101, and the collected tissues were used for genotyping. Prior to beginning the experiments, mice were allowed to acclimate to the experimental rooms for ≥7 days. Mice were randomly assigned to experimental groups, monitored every other day to ensure continuous access to food and water, and animals showing illness or abnormal weight were excluded.

Hypoxic REP cell activation was achieved by exposing mice to 0.1% CO for 4 h. Monitoring during this time included scoring of attitude, respiration, gait and posture. Animals were immediately put back to normal air in the event that accumulated scores exceeded a predefined threshold. Reoxygenation led to immediate recovery, and Hb‐CO was at baseline after ∼1 h (Dahl et al., 2022b).

At the experimental end point, mice were anaesthetized by inhalation of 5% isoflurane in oxygen using a VetFlo vaporizer (Kent Scientific, Torrington, CT, USA). Complete anaesthesia was confirmed by assessing the pedal withdrawal reflex and tail pinch three times. Thereafter, mice were killed by cervical dislocation, and organs were removed.

For UUO, mice were anaesthetized with 5% isofluorane under buprenorphine analgesia (0.1 mg/kg, subcutaneous injection), and were kept under 2.5% isofluorane during the operation. The depth of anaesthesia was monitored as above. The bladder and ureter were exposed through an abdominal incision, and the left ureter was ligated. The peritoneum and skin were closed with single stitch sutures using absorbable suture material and non‐resorbable thread, respectively. Mice were allowed to recover for 3 days at 30°C, receiving buprenorphine during the day (0.1 mg/kg subcutaneously, at least every 6 h) and night (0.01 mg/mL in drinking water). Mice were monitored daily for signs of pain, including hunched posture, poor grooming, reduced mobility, progressive body weight, and water and food uptake. Euthanasia was performed in the event of failure of pain‐mitigating measures and body weight loss of ≥10%. After 7 days, mice were injected intraperitoneally with 50 mg/kg FG‐4592/roxadustat (Selleckchem, Houston, TX, USA), dissolved in 0.5 M Tris–HCl pH 9.0 (20 mg/mL), and after 90 min, mice were anaesthetised and killed as described above.

### 2.3 Spatial transcriptomics

For RNA quality assessment, RNA from 20–30 mg of optimum cutting temperature (OCT)‐embedded tissue was extracted using the RNeasy FFPE kit (Qiagen, Hilden, Germany). The RNA yield and purity were measured using a NanoDrop spectrophotometer (Thermo Fisher Scientific, Waltham, MA, USA), and RNA integrity was assessed via the ScreenTape assay on an automated TapeStation 4200 system (Agilent Technologies, Basel, Switzerland). A minimal fraction of 30% of RNA fragments with >200 nucleotides was considered sufficient for further analysis.

Spatial transcriptomics of mouse kidneys was performed according to the manufacturer's recommendations (Visium CytAssist for fixed frozen tissue; 10× Genomics, Pleasanton, CA, USA). Briefly, cryosections (10 µm thick) were prepared using a cryotome (CM3050S; Leica, Wetzlar, Germany) and placed on SuperFrost Plus adhesion microscope slides (Epredia, Essendonk, The Netherlands). Rehydrated tissues were stained with Haematoxylin and Eosin according to the manufacturer's protocol (10× Genomics) and imaged using a slide scanner with a 20× dry objective (Axio Scan.Z1; Zeiss Microscopy, Oberkochen, Germany). Subsequently, tissues were transferred to Visium slides, followed by permeabilization, reverse transcription, library preparation and sequencing according to the manufacturer's instructions (10× Genomics). A detailed description of the Visium data analyses (GeneVia Technologies, Tampere, Finland) and the spot numbers of all kidney slices analysed are provided in the Appendix and Table S1, respectively.

### 2.4 Messenger RNA fluorescence in situ hybridization

Kidneys were excised, cut in half, and incubated in 4% paraformaldehyde solution overnight at 4°C. Fixed tissues were dehydrated in 30% sucrose and embedded in OCT compound (Tissue‐Tek; Sakura Finetek, Alphen aan den Rijn, The Netherlands). Cryosections (8 µm thick) were prepared as above and pretreated according to the manufacturer's recommendations (RNAscope Multiplex Fluorescent v2 Assay; Advanced Cell Diagnostics, Hayward, CA, USA). RNAscope probes (Table A1) were hybridized for 2 h at 40°C in a HybEZ oven (Advanced Cell Diagnostics), followed by signal amplification and signal detection using Opal 520, 570, 650 or 690 fluorescent reagents (Akoya Biosciences; Marlborough, MA, USA). Fluorescent signals were recorded using a slide scanner (Axio Scan.Z1; Zeiss Microscopy).

### 2.5 Immunofluorescence

Cryosections were blocked and permeabilized with 5% normal goat serum (AB_2336990; Jackson ImmunoResearch, Ely, UK) and 0.3% Triton X‐100 (Sigma–Aldrich, Burlington, MA, USA) in PBS, and incubated with primary antibodies (Table A2) overnight at 4°C. After washing three times in 0.05% Tween‐20 (Sigma–Aldrich) in Tris‐buffered saline, sections were incubated with secondary antibodies for 1 h at room temperature. Nuclei were counterstained with 0.5 µg/mL 4′,6‐diamidino‐2‐phenylindole (DAPI; Sigma–Aldrich). The sections were mounted using ProLong Gold antifade mountant (P36930; Thermo Fisher Scientific), and fluorescence signals were recorded using a slide scanner as above. For sequential immunofluorescence, post‐hybridization sections were treated according to the manufacturer's recommendations (Opal 3‐plex manual detection kit; Akoya Biosciences).

### 2.6 Image quantification

Kidney areas were annotated manually based on tubular autofluorescence, *Epo* mRNA (REP cells) and segmental nephron protein markers. For the quantification of all other protein markers, the machine learning algorithm ‘pixel classifier’ (QuPath v.0.4.0) was used (Bankhead et al., 2017). Briefly, a similar small number of diverse signals were annotated manually using the ‘wand tool’, marking pixels of similar intensities. Those annotations served as training examples for each class. An Artificial Neural Network Multilayer Perceptron (ANN/MLP) type of classifier and full resolution (0.33 µm per pixel) settings were used. A Gaussian filter was applied by default to smooth the signals and remove noise. To determine the distance of annotated *Epo* mRNA‐positive cells to the nearest detected signal of a given marker, a custom script written in Groovy scripting language was applied (see Supp. Java Script provided in the Supporting Information) (Champeau et al., 2015).

### 2.7 Statistical analyses

Statistical analyses were performed using Prism v.10 (GraphPad Software, San Diego, CA, USA). Where appropriate, data were assessed by repeated‐measures two‐way ANOVA, followed by the *post hoc* tests indicated in the figure legends. *P*‐values of <0.05 were considered statistically significant.

## 3 RESULTS

### 3.1 Identification of renal cell types neighbouring REP cells by spatial transcriptomics

**FIGURE 1:**
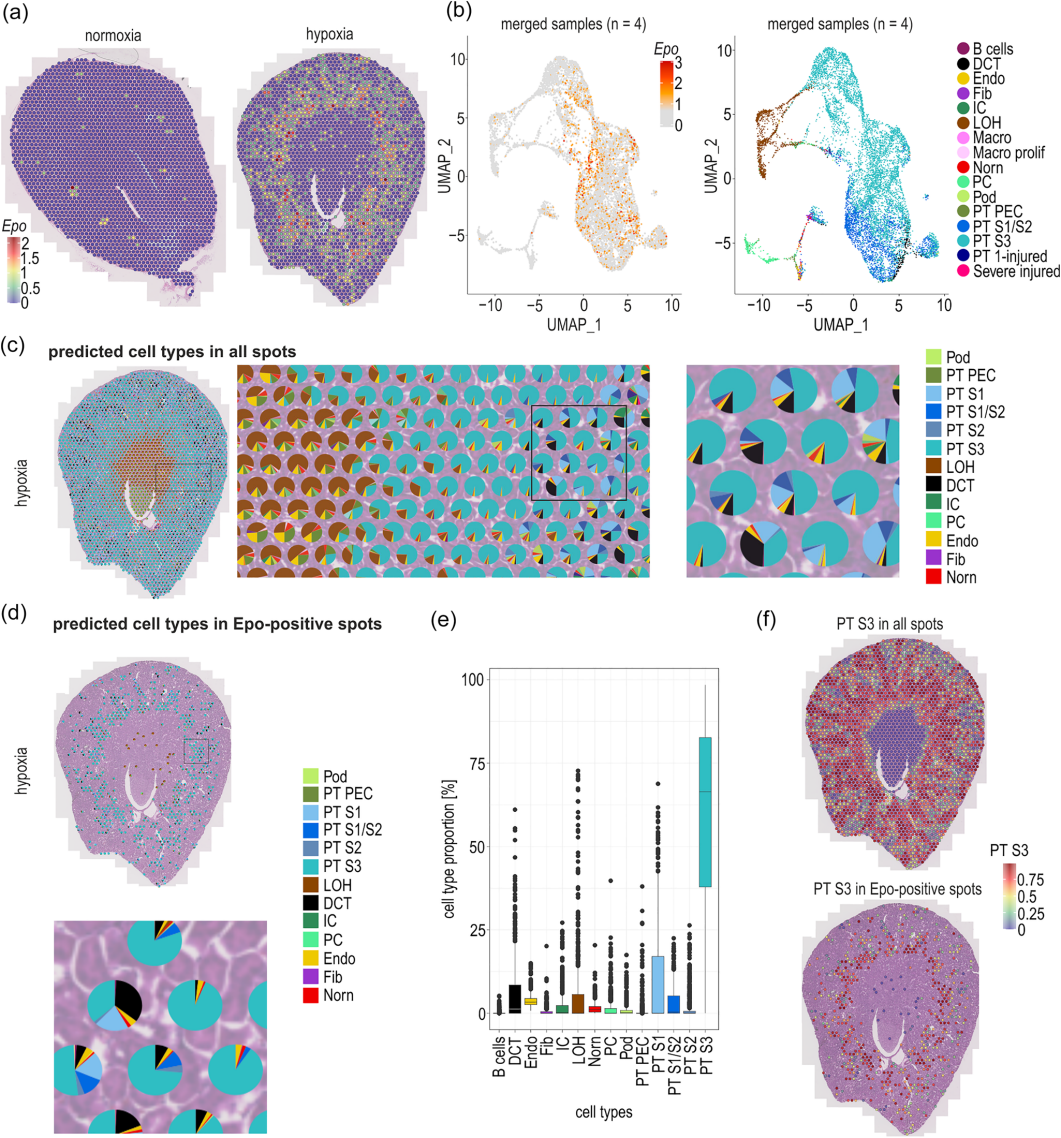
Spatial transcriptomics of normoxic and hypoxic mouse kidneys. (a) Representative Visium spatial plots of kidney samples derived from mice exposed to normoxia or to 0.1% CO for 4 h (hypoxia). *Epo* mRNA levels are represented by standardized values (Pearson residuals) derived from SCTransform normalization. (b) Uniform manifold approximation and projection (UMAP) plots of merged normoxic (*n* = 2; 1 male, 1 female) and hypoxic (*n* = 2; 2 males) samples, indicating *Epo* mRNA levels (left panel) or annotated cell types following Visium spot deconvolution (right panel). (c). Cell type deconvolution of all Visium spots of a representative hypoxic male kidney (background: Haematoxylin and Eosin staining). Pie charts show the estimated cell type proportions inferred from total mRNA expression patterns in each spot, annotated based on reference scRNAseq databases. (d) Cell type deconvolution of *Epo* mRNA‐positive Visium spots. (e) Box plot showing cell type proportions of *Epo* mRNA‐positive Visium spots derived from spot deconvolution. Normalized gene expression values were obtained with SCTransform and are represented as standardized, variance‐stabilized Pearson residuals. (f) PT S3 cell proportions in all or only *Epo* mRNA‐positive Visium spots of a hypoxic kidney, visualized by the indicated colour scale. Abbreviations: 1‐injured, type 1 injury; DCT, distal convoluted tubule; Endo, endothelial cells; Fib, fibroblasts; IC, intercalated cells; LOH, loop of Henle; Macro, macrophages; PC, principal cells; PEC, parietal epithelial cells; Pod, podocytes; prolif, proliferating; PT, proximal tubule; S1/S2/S3, segments 1/2/3; Severe injured, severely injured PT.

To enhance *Epo* mRNA expression in REP cells, mice were exposed to 0.1% carbon monoxide (CO) for 4 h, resulting in 50% CO saturation of haemoglobin (Dahl et al., 2022b). Subsequently, the spatial transcriptome of their kidneys was investigated by Visium technology. Each Visium spot comprises all mRNA species within a diameter of 55 µm, allowing for the identification of the direct neighbourhood of REP cells. When compared with normoxic control kidneys, a strong hypoxic increase in *Epo* mRNA spot intensity was observed (Figure 1a), enriched in the corticomedullary border regions, in a similar manner to the previously reported direct detection of *Epo* mRNA by in situ hybridization (Broeker et al., 2020; Dahl et al., 2022b; Firmke et al., 2025; Imeri et al., 2019).

Dimensionality of merged data from normoxic and hypoxic samples was reduced by applying principal component analysis (PCA) based on 30 principal components (PCs). The resulting uniform manifold approximation and projection (UMAP) plots represent transcriptional similarity amongst spatial spots across samples, with clusters visualized based on shared gene expression profiles. Despite prominent *Epo* mRNA in Visium spots, there was no obvious clustering of spots containing REP cells (Figure 1b, left panel). Although REP cells were dispersed across Visium spots with a highly diverse cell composition, there was a certain overlap with spots high in mRNA specific to tubular epithelial cells of the cortex and outer medulla [PT and distal convoluted tubule (DCT)], whereas other cell types [intercalated cells (IC), loop of Henle (LOH) and principal cells (PC)] showed less overlap (Figure 1b, right panel).

To estimate the cell type proportions contributing to each Visium spot, we aligned RNA sequencing counts with custom annotated reference datasets and computed probabilistic cell type proportions and weights as described in the Appendix (Kirita et al., 2020; Kragesteen et al., 2023; Lake et al., 2023; Wang et al., 2022), assuming an unknown mixture of multiple cells (Cable et al., 2022). Consistent with a previous report showing that the contribution of PT cell‐derived mRNA to total mRNA in whole kidney transcriptomic data was >50% (Clark et al., 2019), our cell type deconvolution confirmed the high proportion of PT cell‐positive spots (Figure 1c). The global predominance of PT markers in the cortex and outer medulla, and LOH markers in the inner medulla, confirmed the correct cell type assignment and deconvolution. In line with the absence of a clearly defined *Epo*‐positive cluster in UMAP plots, Norn cells represented only a minor, if any, fraction in Visium spots (Figure 1c, right panel).

**FIGURE A1:**
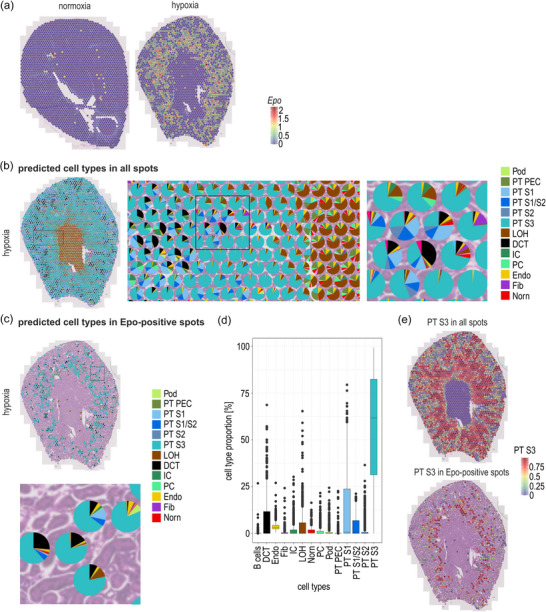
Spatial transcriptomics of normoxic and hypoxic mouse kidneys. Reproduction of the experiment shown in Figure 1 in kidneys derived from independent mice. (a) Visium spatial plots of kidney samples derived from mice exposed to normoxia (*n* = 1; male) or to 0.1% CO (*n* = 1; male) for 4 h (hypoxia). *Epo* mRNA levels are indicated by standardized values (Pearson residuals) after normalization by SCTransform. (b) Cell type deconvolution of all Visium spots of the replicant hypoxic kidney (background: Haematoxylin and Eosin staining). Pie charts show the estimated cell type proportions inferred from total mRNA expression patterns in each spot, annotated based on reference scRNAseq databases. (c) Cell type deconvolution of *Epo* mRNA‐positive Visium spots. (d) Box plot showing cell type proportions of *Epo* mRNA‐positive Visium spots. Scaled values represent standardized and variance‐stabilized expression values (Pearson residuals). (e) Proximal tubule (PT) segment 3 (S3) cell proportions in all or only *Epo* mRNA‐positive Visium spots in replicant hypoxic kidney, visualized by the indicated colour scale. Abbreviations: DCT, distal convoluted tubule; Endo, endothelial cells; Fib, fibroblasts; IC, intercalated cells; LOH, loop of Henle; PC, principal cells; PEC, parietal epithelial cells; Pod, podocytes; PT, proximal tubule; S1/S2/S3, segments 1/2/3.

As confirmed by restricting the further analysis to *Epo* mRNA‐positive spots in hypoxic kidneys, Norn cells remained a minor fraction even in these spots (Figure 1d), which are dominated by the abundance of PT S3 cells, followed by endothelial cells (Figure 1e). Although this result might imply that PT S3 cells are preferentially neighbouring REP cells, cell type deconvolution based on mRNA abundance is highly influenced by cell size (i.e., mRNA content) and density. Indeed, the large and abundant PT S3 cells dominate most Visium spots in the cortex and outer medulla (Figure 1f, upper panel), and all Epo mRNA‐positive spots in these regions are PT S3 positive (Figure 1f, lower panel). Similar Visium data were obtained with independent kidney samples derived from a second normoxic and a second hypoxic mouse (Figure A1), which were included in the merged UMAP plot shown in Figure 1b.

### 3.2 Proximity of REP cells to nephron segments positive for Sglt2 and Sglt1

**FIGURE 2:**
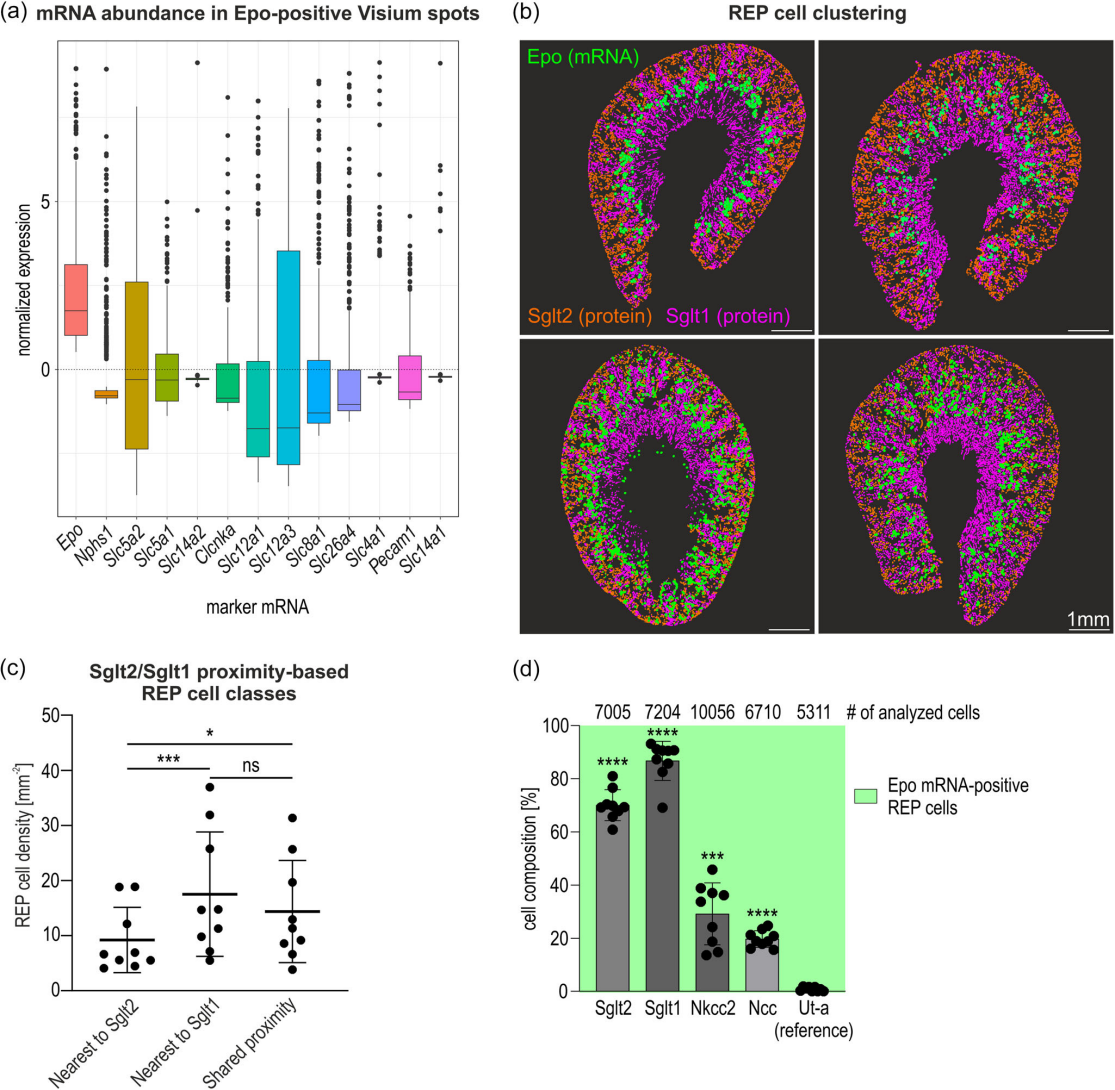
Nephron segment‐specific localization of REP cells in hypoxic mouse kidneys. (a) Box plots showing standardized Pearson residuals (mean = 0, SD = 1) representing abundance of *Epo* and the indicated nephron segment‐specific marker mRNAs in *Epo*‐positive Visium spots relative to all Visium spots in a hypoxic male mouse kidney. A value higher than zero‐ indicates above‐average expression compared with all Visium spots. (b) Combined detection of *Epo* (mRNA‐FISH) and Sglt2/Sglt1 (sequential immunofluorescence) in independent hypoxic kidney samples (*n* = 4; 2 males, 2 females). For better visibility, positive cells are highlighted artificially. Scale bar: 1 mm. (c) REP cell density in either of the three indicated classes based on the distance between *Epo* mRNA‐FISH and Sglt2/1 immunofluorescence signals. ‘Shared proximity’ was assigned when both Sglt2 and Sglt1 were within 15 µm distance; REP cells beyond 15 µm from either Sglt2 or Sglt1 were ignored. Shown are mean values ± SD of *n* = 9 (average of two sections per mouse; 5 males, 4 females) independent hypoxic kidneys. One‐way ANOVA with repeated‐measures followed by Bonferroni's multiple comparisons test was used to evaluate the differences between the three classes statistically. *P = 0.0116; ***P = 0.0002; ns, not significant, *P* = 0.1538. (d) Percentage of the indicated marker protein signals within 15 µm distance from *Epo* mRNA signals. The numbers of analysed *Epo* mRNA‐positive REP cells are listed on top. Shown are mean values ± SD of *n* = 9 (average of two sections per mouse; 5 males, 4 females) independent hypoxic kidneys. One‐way ANOVA followed by Dunnett's *post hoc* correction was used to evaluate the differences between the cortical/outer medullary (Sglt2/1, Nkcc2 and Ncc) and the inner medullary reference (Ut‐a) markers statistically. ***P = 0.0002; ****P < 0.0001.

**FIGURE A2:**
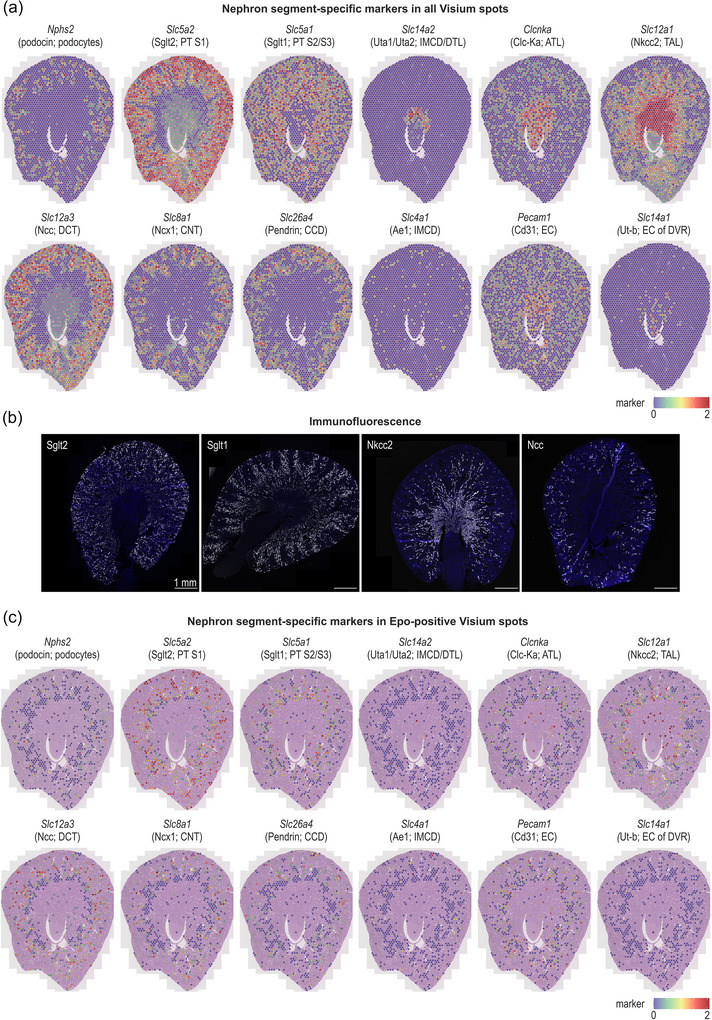
Nephron segment‐specific localization of REP cells by spatial transcriptomics. (a, c) Abundance of the indicated nephron segment‐specific marker mRNAs in all Visium spots (a) or in *Epo* mRNA‐positive spots only (c) of a hypoxic mouse kidney (*n* = 1; male). Marker mRNA levels are indicated by standardized values (Pearson residuals) after normalization by SCTransform. (b) Immunofluorescence confirmation of the indicated nephron segment‐specific marker proteins (white) in independent kidney samples (*n* = 4; 3 males, 1 female). Nuclei were stained with DAPI (blue). Scale bar: 1 mm. Abbreviations: ATL, ascending thin limb; CCD, cortical collecting duct; CNT, connecting tubule; DCT, distal convoluted tubule; DTL, descending thin limb; DVR, descending vasa recta; EC, endothelial cells; IMCD, inner medullary collecting duct; PT, proximal tubule; S1/S2/S3, segments 1/2/3; TAL, thick ascending limb.

**FIGURE A3:**
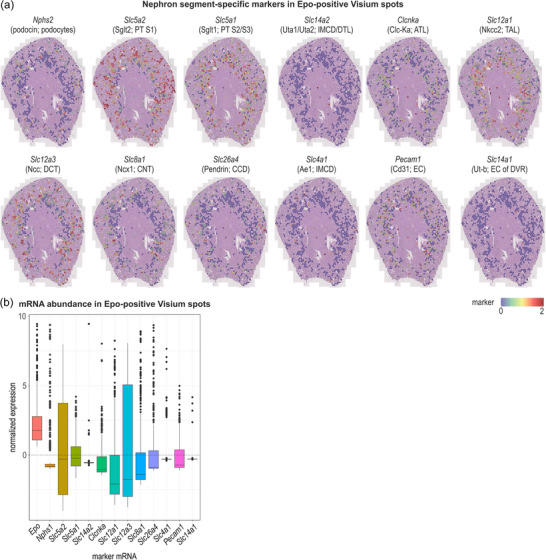
Nephron segment‐specific localization of REP cells by spatial transcriptomics of an independent kidney. (a) Reproduction of the experiment shown in Figures 2 and A2 in an independent hypoxic male mouse kidney. (b) Box plot of standardized Pearson residuals (mean = 0, SD = 1) representing the abundance of *Epo* and the indicated nephron segment‐specific marker mRNAs in *Epo* mRNA‐positive Visium spots relative to all Visium spots. A value higher than zero indicates above‐average expression compared with all Visium spots. Abbreviations: ATL, ascending thin limb; CCD, cortical collecting duct; CNT, connecting tubule; DCT, distal convoluted tubule; DTL, descending thin limb; DVR, descending vasa recta; EC, endothelial cells; IMCD, inner medullary collecting duct; PT, proximal tubule; S1/S2/S3, segments 1/2/3; TAL, thick ascending limb.

To refine the REP cell neighbourhood analysis, we used a panel of segment‐specific nephron markers (Agarwal et al., 2021) and quantified their mRNA abundance in all Visium spots of a hypoxic kidney sample (Figure A2a). Immunofluorescence analysis of independent kidneys confirmed the global distribution of the markers Sglt2 (*Slc5a2*; PT S1), Sglt1 (*Slc5a1*; PT S2/S3), Nkcc2 (*Slc12a1*; TAL) and Ncc (*Slc12a3*; DCT) at the protein level (Figure A2b). We then restricted the analysis to *Epo* mRNA‐positive Visium spots only (Figure A2c). Upon SCTransform‐based normalization, *Epo* was the most abundant mRNA species in these spots, followed by similar levels of the PT markers *Slc5a1* (Sglt1) and *Slc5a2* (Sglt2) and the endothelial cell marker *Pecam1* (Cd31) (Figure 2a). In contrast, markers for distal parts of the nephron, such as *Slc12a1* (Nkcc2) and *Slc12a3* (Ncc), were scarce. Several other markers that also showed high normalized expression levels (*Slc14a2*, *Slc4a1* and *Slc14a1*) were not considered because of their presence in only very low numbers of Visium spots. Essentially identical data were obtained with a kidney sample derived from an independent hypoxic mouse (Figure A3).

**FIGURE A4:**
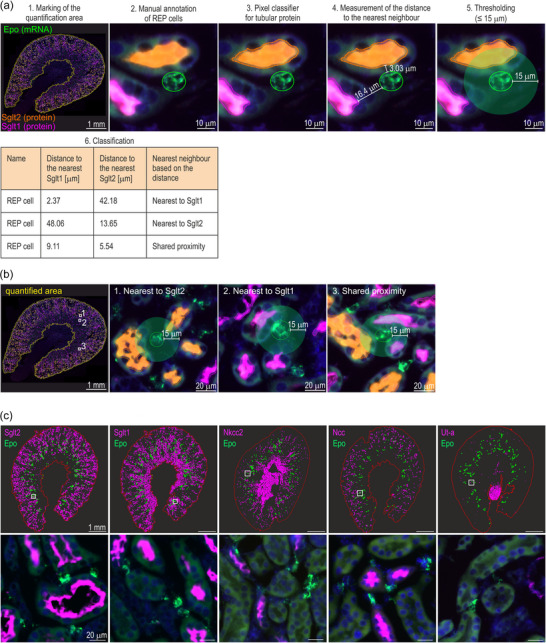
Distance measurements between *Epo* mRNA and Sglt2/Sglt1 protein. (a) Workflow of the steps used for measuring the distance between *Epo* mRNA‐FISH (green) and Sglt2/Sglt1 sequential immunofluorescence signals (orange and magenta, respectively). The quantification area was delineated based on autofluorescence and protein signals. Sglt2/Sglt1‐negative areas (inner stripe of outer medulla and inner medulla) were excluded from the analysis. REP cells were annotated manually. Sglt2 and Sglt1 protein signals were detected by QuPath pixel classifier, and a custom script was used to calculate distances. Only distances ≤15 µm were considered, and ‘shared proximity’ was classified when both Sglt2 and Sglt1 were within this threshold. (b) Representative examples of REP cells classified as ‘nearest to Sglt2’, ‘nearest to Sglt1’ or ‘shared proximity’. (c) Representative examples of additional proximity analyses between Epo mRNA (green) and markers of the thick ascending limb (Nkcc2 and *Slc12a1*), distal convoluted tubule (Ncc and *Slc12a3*) and inner medullary collecting duct (Ut‐a and *Slc14a2*) (magenta). Scale bars: 1 mm in upper panel; 20 µm in lower panel.

As visualized by *Epo* mRNA‐FISH, REP cells are not randomly distributed but appear to be grouped into small clusters, spatially separated by the nephron segments positive for either Sglt2 or Sglt1 protein (Figure 2b). Therefore, we wondered whether there is any spatial preference of REP cells for a PT segment‐specific neighbourhood. According to the Visium spot analysis, there was a similar abundance of *Slc5a2* (Sglt2) and *Slc5a1* (Sglt1) mRNA in the *Epo*‐positive spots. However, the low spatial resolution of this technique does not allow for drawing any conclusion about the exact proximity, or lack thereof, of REP cells to either Sglt2‐ or Sglt1‐positive PT cells. We therefore measured the distance of REP cells (cytoplasmic *Epo* mRNA‐FISH signals) from either PT S1 or S2/S3 (luminal brush border Sglt2 or Sglt1 sequential immunofluorescence signals, respectively), as illustrated in Figure A4a, b. ‘Shared proximity’ was classified when both Sglt2 and Sglt1 signals were within 15 µm, equal to half of the average diameter of a mouse PT (Morozov et al., 2021). Signals >15 µm distant from REP cells were considered too distal and therefore excluded from the analysis. These measurements revealed a significantly higher REP cell density nearer to Sglt1 or to both Sglt1 and Sglt2 (shared proximity) than to Sglt2 alone (Figure 2c; Table S2). When calculating the percentage of the immunofluorescence signals <15 µm distant from *Epo* mRNA‐FISH signals, 70.1% and 86.8% of REP cells were proximal to Sglt2 and Sglt1, respectively (Figure 2d; Table S2). In comparison, only 29.2%, 19.7% and 0.8% of REP cells were closest to markers of the thick ascending limb (Nkcc2, *Slc12a1*), distal convoluted tubule (Ncc, *Slc12a3*) and inner medullary collecting duct (Ut‐a, *Slc14a2*) (Shayakul et al., 2013), respectively (Figures 2d and A4c; Table S2).

In summary, these data demonstrate that REP cells are most closely located to endothelial cells on the interstitial side and to PT epithelia on the tubular side, with a certain preference for PT S2/S3 segments, both of which might have functional implications for REP cell differentiation and/or function.

### 3.3 Pharmaceutical Epo reinduction in REP cells with a heavily damaged tubular neighbourhood

To investigate the cellular changes around REP cells during CKD, we analysed damaged kidneys following 7 days of UUO and compared them with healthy contralateral kidneys. We have previously shown that the ligated but not contralateral kidney lost Epo production and that after a brief 90 min treatment with FG‐4592/roxadustat, Epo was again expressed in the ligated kidney to a similar extent to the contralateral kidney (Dahl et al., 2022b). However, the extent to which the immediate tubular neighbourhood of these REP cells was affected by the UUO‐induced injury remained unclear.

**FIGURE 3:**
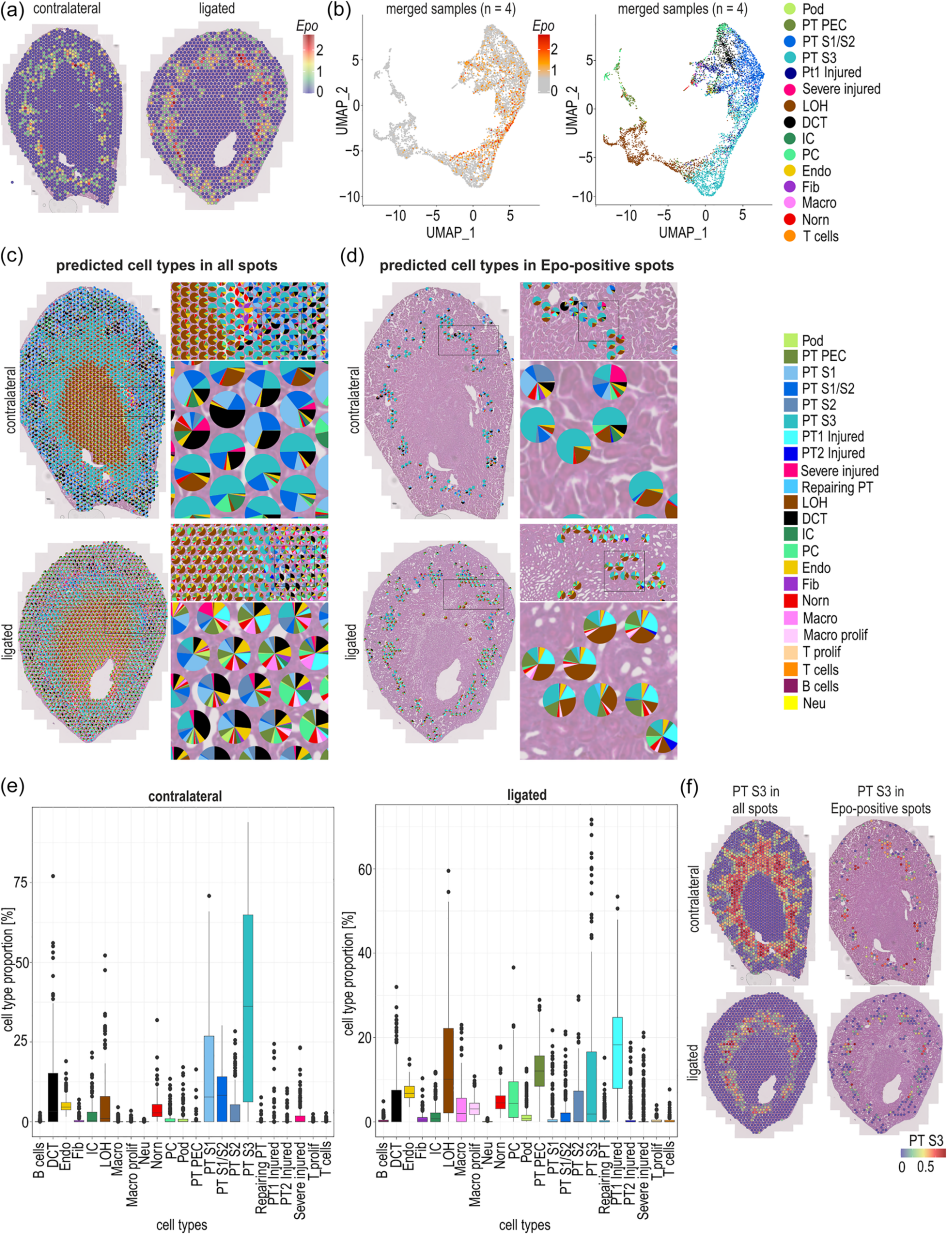
Spatial transcriptomics of injured mouse kidneys treated with roxadustat. (a) Representative Visium spatial plots of contralateral and ligated kidneys, following 7 days of unilateral ureteral obstruction and 90 min treatment with FG‐4592/roxadustat. *Epo* mRNA expression levels are indicated by standardized values (Pearson residuals derived from SCTransform normalization). The colour scale (key) represents Pearson residuals. (b) UMAP plots of merged contralateral (*n* = 2; 2 males) and ligated (*n* = 2; 2 males) kidneys, indicating *Epo* mRNA levels (left panel) or annotated cell types following Visium spot deconvolution (right panel). (c) Cell type deconvolution of all Visium spots of representative contralateral and ligated kidneys (2 males; background: Haematoxylin and Eosin staining). Pie charts show the estimated cell type proportions inferred from total mRNA expression patterns in each spot, annotated based on a reference scRNAseq database. (d) Cell type deconvolution of *Epo* mRNA‐positive Visium spots. (e) Box plots showing cell type proportions of *Epo* mRNA‐positive Visium spots derived from spot deconvolution. Normalized gene expression values were obtained with SCTransform and are represented as standardized, variance‐stabilized Pearson residuals (*n* = 1; male). (f) Proximal tubule segment 3 (PT S3) proportions in all or only *Epo* mRNA‐positive Visium spots in contralateral and ligated samples, visualized by the indicated colour scale. Abbreviations: CD, collecting duct; CT, connecting tubule; DCT, distal convoluted tubule; DTL‐ATL, descending/ascending thin limb; Endo, endothelial cells; Fib, fibroblasts; IC, intercalated cells; ICA‐ICB, IC type A/B; IMCD, inner medulla collecting duct; LOH, loop of Henle; Macro, macrophages; Myofib, myofibroblasts; Neu, neutrophils; PC, principal cells; PEC, parietal epithelial cells; Pod, podocytes; prolif, proliferating; PT, proximal tubule; S1/S2/S3, segments 1/2/3; Uro, urothelium.

**FIGURE A5:**
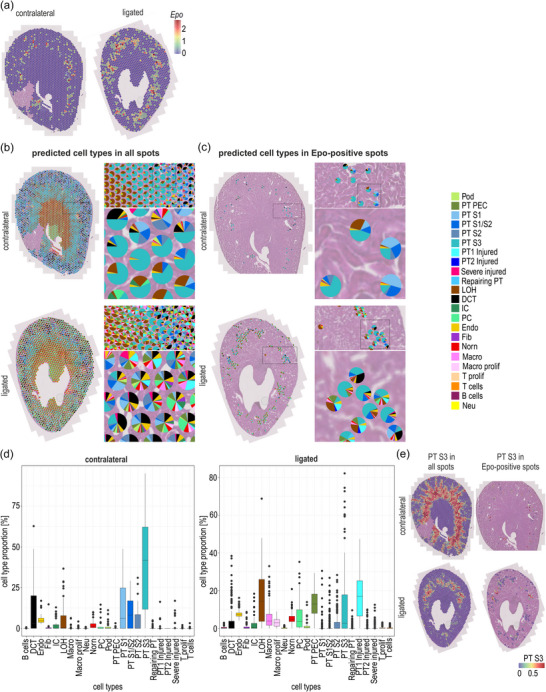
Spatial transcriptomics of injured mouse kidneys treated with roxadustat. Reproduction of the experiment shown in Figure 3 in kidneys derived from an independent male mouse after 7 days of unilateral ureteral obstruction and 90 min of treatment with FG‐4592/roxadustat. (a) Representative Visium spatial plots of contralateral and ligated kidneys. *Epo* mRNA expression levels are indicated by standardized values (Pearson residuals) after normalization by SCTransform. Colour scale (key) represents Pearson residuals. (b) Cell type deconvolution of all Visium spots of contralateral and ligated kidneys (background: Haematoxylin and Eosin staining). Pie charts show the estimated cell type proportions inferred from total mRNA expression patterns in each spot, annotated based on a reference scRNAseq database. (c) Cell type deconvolution of *Epo* mRNA‐positive Visium spots. (d) Box plots showing cell type proportions of *Epo* mRNA‐positive Visium spots. Scaled values represent standardized, variance‐stabilized expression values (Pearson residuals). (e) Proximal tubule segment 3 (PT S3) proportions in all or only *Epo* mRNA‐positive Visium spots in contralateral and ligated samples, visualized by the indicated colour scale. Abbreviations: DCT, distal convoluted tubule; Endo, endothelial cells; Fib, fibroblasts; IC, intercalated cells; LOH, loop of Henle; Macro, macrophages; Neu, neutrophils; PC, principal cells; Pod, podocytes; prolif, proliferating; PT, proximal tubule; S1/S2/S3, segments 1/2/3.

Spatial transcriptomics of the contralateral and ligated kidneys derived from the same mouse confirmed both the similar strength and the preserved localization of *Epo* mRNA reinduction following roxadustat treatment (Figure 3a). UMAP plots of merged data derived from contralateral and ligated kidney samples (Figure 3b, left panel) showed *Epo* mRNA induction in Visium spots similar to hypoxic healthy mice (Figure 1b, left panel). Again, *Epo* mRNA‐positive Visium spots did not cluster and were dispersed across a highly diverse cell composition (Figure 3b, right panel). As expected, spot deconvolution revealed extensive changes in cell composition between spots of the ligated kidney compared with the contralateral one (Figure 3c). Focusing on *Epo* mRNA‐positive Visium spots, there was a major shift from healthy to injured PT cell types in the REP cell neighbourhood (Figure 3d). Although PT S3 followed by S1/S2 and S1 cells were most prominently represented in *Epo*‐positive spots of the contralateral kidney, ‘PT1 injured’ followed by parietal epithelial and LOH cells were the main neighbours of REP cells in the ligated kidney (Figure 3e). The loss of PT S3 cells in the REP cell vicinity is attributable mainly to a general loss of this cell type in the ligated kidney (Figure 3f). There was also a strong increase in two macrophage populations, suggesting immune cell infiltration, including in the vicinity of REP cells. Of note, the proportions of endothelial cells and Norn cells remained stable in the *Epo* mRNA‐positive spots of the damaged kidneys (Figure 3e). Similar Visium data were obtained with independent contralateral and ligated kidney samples derived from a second mouse treated with roxadustat after 7 days of UUO (Figure A5), which were included in the merged UMAP plot shown in Figure 3b.

### 3.4 Preferential Epo reinduction in REP cells proximal to injured PT cells with maintained Sglt1 expression

**FIGURE 4:**
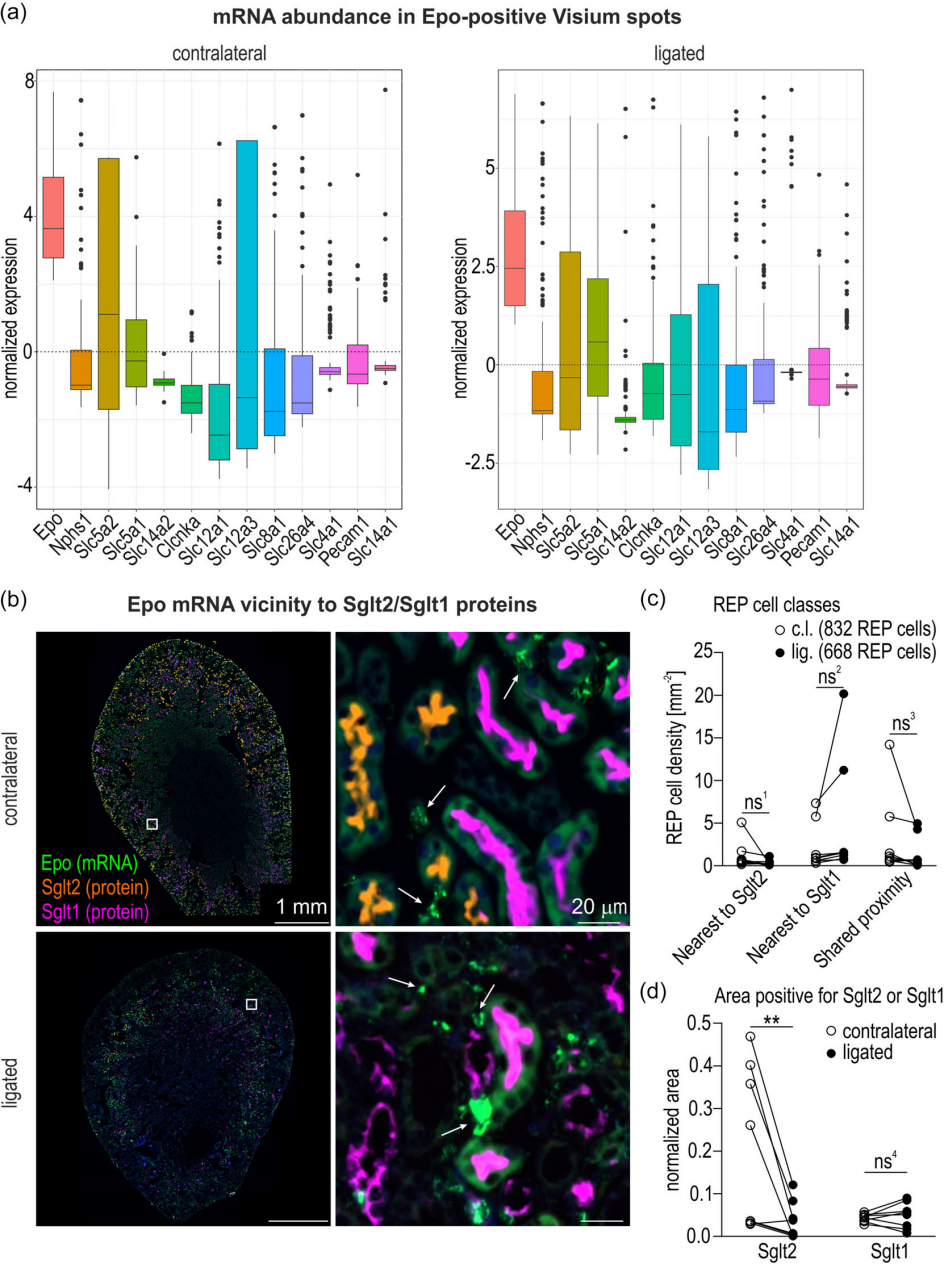
Nephron segment‐specific localization of REP cells in injured mouse kidneys. (a) Box plots showing standardized Pearson residuals (mean = 0, SD = 1) representing abundance of *Epo* and the indicated nephron segment‐specific marker mRNAs in *Epo* mRNA‐positive Visium spots relative to all Visium spots in contralateral and ligated mouse kidneys after 7 days of unilateral ureteral obstruction. A value higher than zero indicates above‐average expression compared with all Visium spots (*n* = 1; male). (b) Combined detection of *Epo* (mRNA‐FISH; indicated by arrows) and Sglt2/Sglt1 (sequential immunofluorescence) in the corresponding contralateral and ligated mouse kidneys. (c) REP cell densities in contralateral (c.l.) and ligated (lig.) kidneys in either of the three indicated classes based on the distance between *Epo* mRNA‐FISH and Sglt2/1 immunofluorescence signals. ‘Shared proximity’ was assigned when both Sglt2 and Sglt1 were within 15 µm distance; REP cells beyond 15 µm from either Sglt2 or Sglt1 were ignored. The numbers of analysed REP cells are indicated (*n* = 8; 5 males, 3 females; average of two sections per mouse). (d) Quantification of total Sglt2/1‐positive protein areas. Normalized values indicate summed areas of each QuPath classifier object, divided by the whole kidney area. (c, d) Repeated‐measures two‐way ANOVA with multiple comparisons followed by Bonferroni's *post hoc* test was used to evaluate the differences in Sglt2 and Sglt1 areas between contralateral and ligated kidneys statistically. ns, not significant; ns^1^, *P* = 0.8683; ns^2^, *P* = 0.1392; ns^3^, *P* = 0.4885; ns^4^, *P* > 0.9999; **P = 0.0023.

**FIGURE A6:**
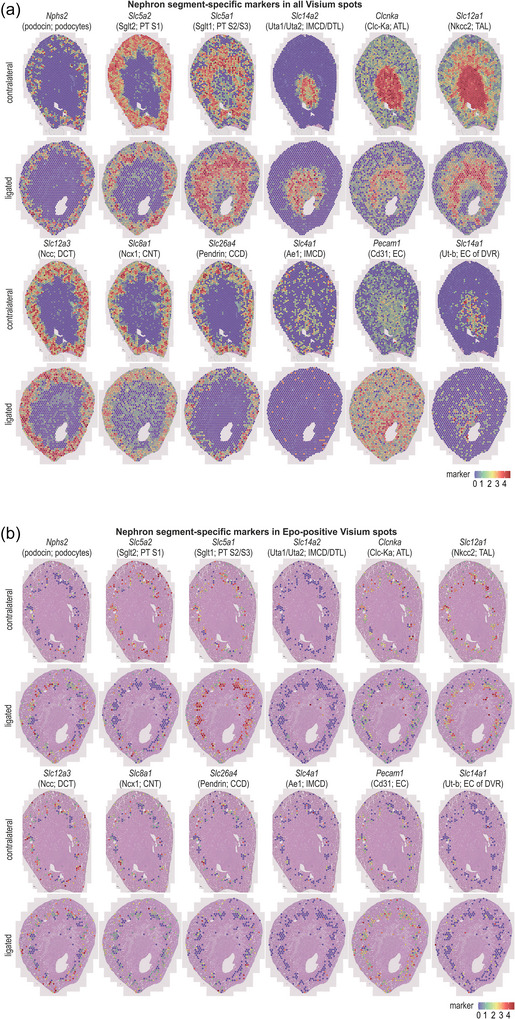
Nephron segment‐specific localization of REP cells by spatial transcriptomics of an injured mouse kidney. Abundance of the indicated nephron segment‐specific marker mRNAs in all Visium spots (a) or in *Epo* mRNA‐positive spots only (b) of contralateral or ligated male mouse kidneys treated with roxadustat following 7 days of unilateral ureteral obstruction. Marker mRNA levels are indicated by standardized values (Pearson residuals) after normalization by SCTransform. Abbreviations: ATL, ascending thin limb; CCD, cortical collecting duct; CNT, connecting tubule; DCT, distal convoluted tubule; DTL, descending thin limb; DVR, descending vasa recta; EC, endothelial cells; IMCD, inner medullary collecting duct; PT, proximal tubule; S1/S2/S3, segments 1/2/3; TAL, thick ascending limb.

**FIGURE A7:**
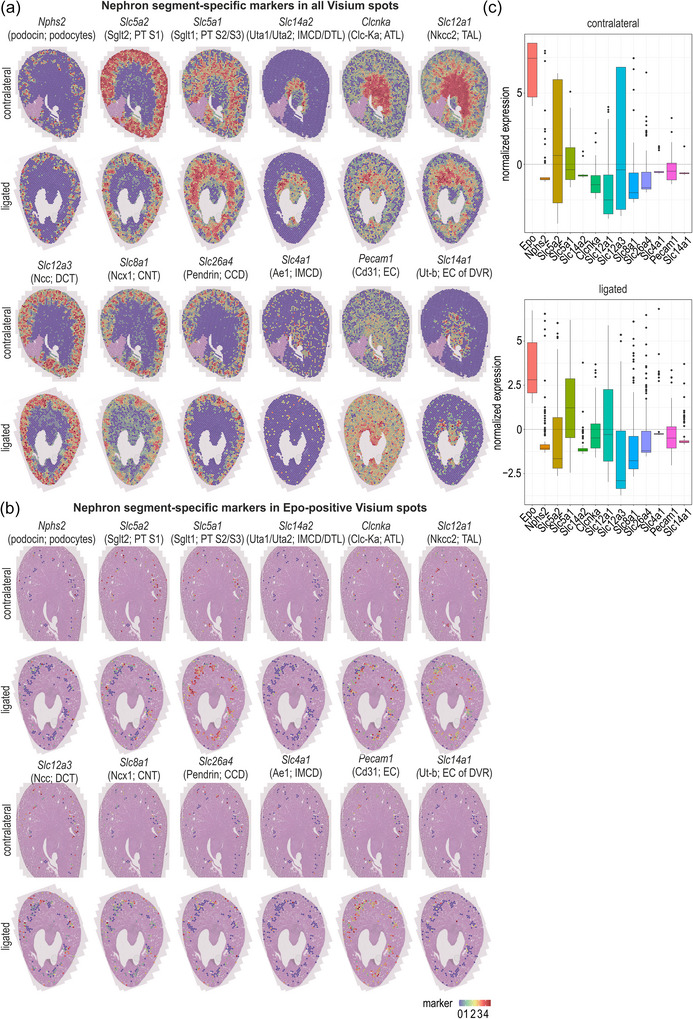
Nephron segment‐specific markers in the vicinity of REP cells identified by spatial transcriptomics. Reproduction of the experiment shown in Figures 4 and A6 in contralateral and ligated kidneys derived from an independent male mouse treated with roxadustat following 7 days of unilateral ureteral obstruction. (a, b) Abundance of the indicated nephron segment‐specific markers mRNAs in all (a) and *Epo* mRNA‐positive (b) Visium spots. Marker mRNA levels are indicated by standardized values (Pearson residuals) after normalization by SCTransform. (c) Box plots of the contralateral and ligated kidneys showing standardized Pearson residuals (mean = 0, SD = 1) representing the abundance of *Epo* and the indicated nephron segment‐specific marker mRNAs in *Epo* mRNA‐positive Visium spots relative to all Visium spots. A value higher than zero indicates above‐average expression compared with all Visium spots. Abbreviations: ATL, ascending thin limb; CCD, cortical collecting duct; CNT, connecting tubule; DCT, distal convoluted tubule; DTL, descending thin limb; DVR, descending vasa recta; EC, endothelial cells; IMCD, inner medullary collecting duct; PT, proximal tubule; S1/S2/S3, segments 1/2/3; TAL, thick ascending limb.

As outlined above for healthy mice, we quantified the abundance of a panel of segment‐specific nephron markers (Agarwal et al., 2021) in all (Figure A6a) or in *Epo* mRNA‐positive (Figure A6b) Visium spots of the kidneys derived from UUO‐injured mice treated with roxadustat. After normalization, *Epo* was the most abundant mRNA species in the *Epo* mRNA‐positive spots of both contralateral and ligated kidneys (Figure 4a), in a similar manner to hypoxic samples of healthy mice (Figures 2a and A3b). However, whilst *Slc5a2* (Sglt2; PT S1) and *Slc5a1* (Sglt1; PT S2/S3) were the next highest mRNA species (similar levels but both below average) in these spots of healthy hypoxic kidneys (Figures 2a and A3b), there was a switch from *Slc5a2* to *Slc5a1* (levels above average) when comparing the contralateral with the ligated roxadustat‐treated kidneys (Figure 4a). This Sglt2‐to‐Sglt1 shift was confirmed by sequential immunofluorescence (Figure 4b, c; Table S3), which demonstrated that the Sglt2 loss was attributable mainly to a general loss of Sglt2, whereas Sglt1 remained constantly expressed (Figure 4d; Table S3). A similar Sglt2‐to‐Sglt1 shift was also found in the second UUO mouse analysed (Figure A7).

**FIGURE 5:**
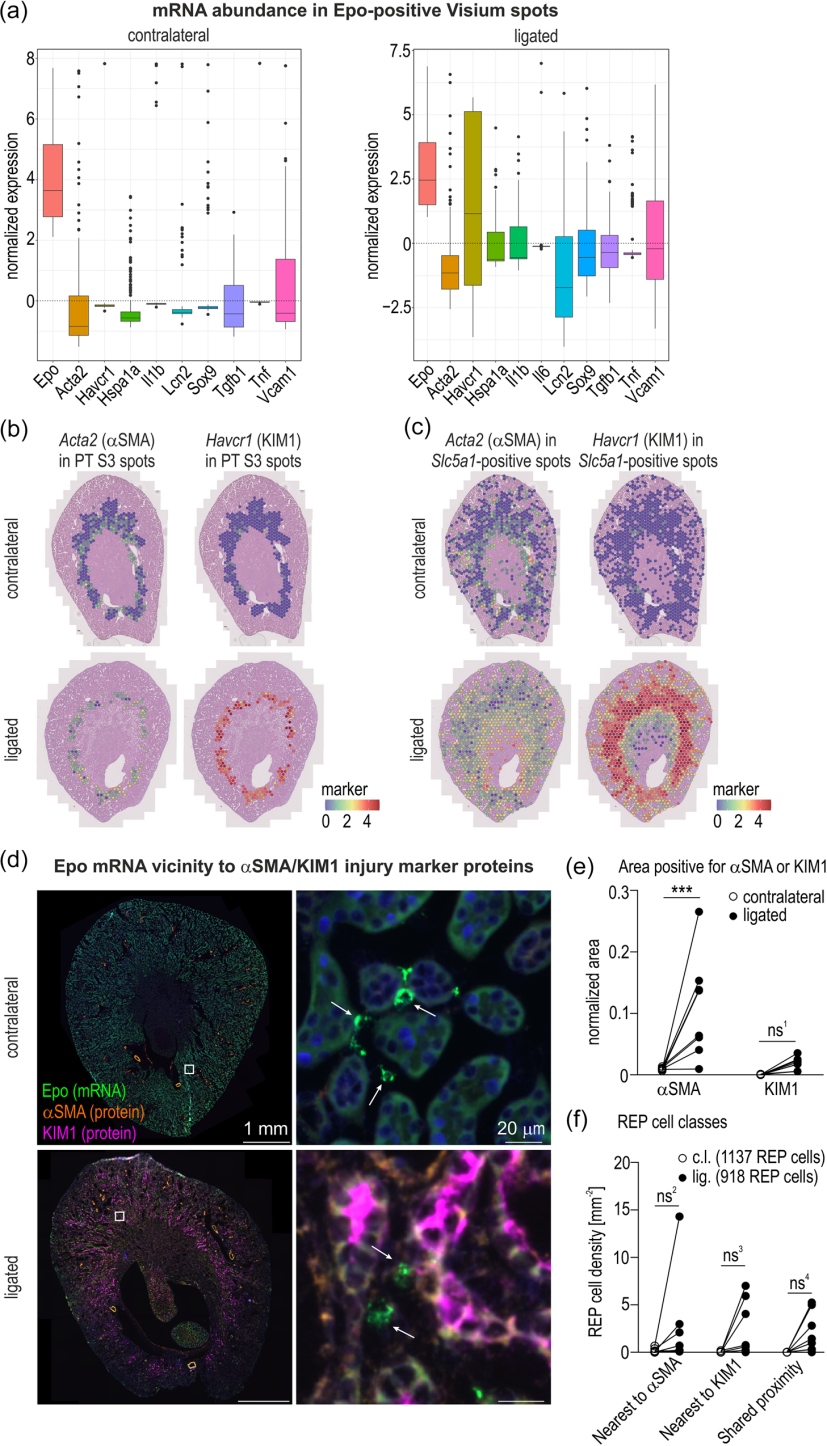
Injury markers in the vicinity of REP cells. (a) Box plots showing standardized Pearson residuals (mean = 0, SD = 1) representing abundance of *Epo* and the indicated kidney injury marker mRNAs in *Epo* mRNA‐positive Visium spots relative to all Visium spots in contralateral and ligated mouse kidneys after 7 days of unilateral ureteral obstruction. A value higher than zero indicates above‐average expression compared with all Visium spots (*n* = 1; male). (b) Levels of the *Acta2* (αSMA) and *Havcr1* (KIM1) injury marker mRNAs in spots where PT S3 cells (based on cell type deconvolution) represent the most abundant cell type, indicated by standardized values (Pearson residuals) after normalization by SCTransform. (c) Levels of *Acta2* and *Havcr1* mRNAs in spots positive for *Slc5a1* (Sglt1). (d) Combined detection of *Epo* (mRNA‐FISH; indicated by arrows) and α‐smooth muscle actin (αSMA) and kidney injury molecule 1 (KIM1) proteins (sequential immunofluorescence) in the corresponding contralateral and ligated mouse kidneys. (e) Quantification of total αSMA and KIM1‐positive protein areas in contralateral and ligated kidneys (*n* = 8; 5 males, 3 females; average of two sections per mouse). Normalized values indicate summed areas of each QuPath classifier object, divided by the whole kidney area. (f) REP cell density in one of the three indicated classes based on the distance between *Epo* mRNA‐FISH and αSMA and KIM1 immunofluorescence signals. ‘Shared proximity’ was assigned when both αSMA and KIM1 were within 15 µm distance; REP cells beyond 15 µm from either αSMA or KIM1 were ignored. The numbers of analysed REP cells are indicated. (e, f) Repeated‐measures two‐way ANOVA with multiple comparisons followed by Bonferroni's *post hoc* test was used to evaluate the differences of αSMA and KIM1 areas between contralateral and ligated kidneys statistically. ***P = 0.0006; ns, not significant; ns^1^, *P* = 0.7795; ns^2^, *P* = 0.1630; ns^3^, *P* = 0.2081; ns^4^, *P* = 0.3265.

**FIGURE A8:**
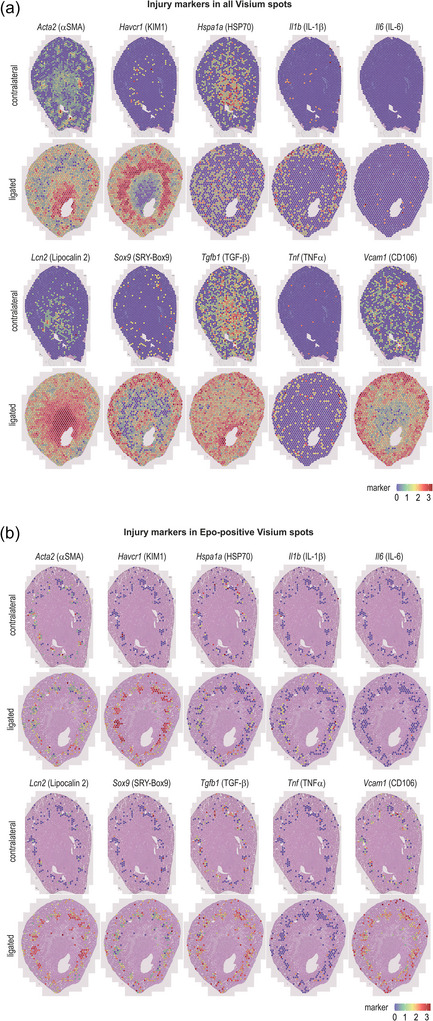
Injury markers in the vicinity of REP cells identified by spatial transcriptomics of an injured mouse kidney. Abundance of the indicated injury marker mRNAs in all (a) and *Epo* mRNA‐positive (b) Visium spots of contralateral or ligated male mouse kidneys treated with roxadustat following 7 days of unilateral ureteral obstruction. Marker mRNA levels are indicated by standardized values (Pearson residuals) after normalization by SCTransform.

**FIGURE A9:**
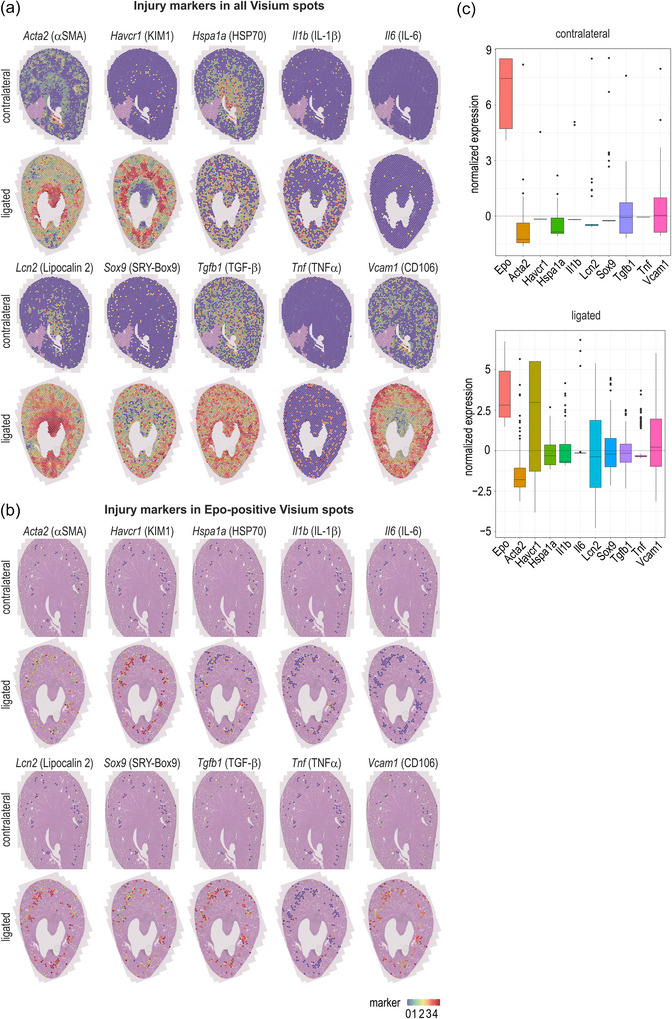
Injury markers in the vicinity of REP cells identified by spatial transcriptomics of an independent injured mouse kidney. Reproduction of the experiment shown in Figures 5 and A8 in contralateral and ligated kidneys derived from an independent male mouse treated with roxadustat following 7 days of unilateral ureteral obstruction. (a, b) Abundance of the indicated injury marker mRNAs in all (a) and *Epo* mRNA‐positive (b) Visium spots. (c) Box plots of the contralateral and ligated kidneys showing standardized Pearson residuals (mean = 0, SD = 1) representing the abundance of *Epo* and the indicated injury marker mRNAs in *Epo* mRNA‐positive Visium spots relative to all Visium spots. A value higher than zero indicates above‐average expression compared with all Visium spots.

Because the majority of the PT S3 cells appeared to be converted to an injured cell type, but the Sglt1 S2/S3 marker was still present in the REP cell vicinity of the ligated kidney, we wondered whether these Sglt1‐positive cells resided in a normal or damaged environment. Therefore, the mRNA abundance of a panel of injury (*Havcr1* and *Lcn2*), (myo)fibrosis (*Acta2* and *Tgfb1*), inflammatory (*Il1b*, *Il6*, *Tnf* and *Vcam1*) and repair/progenitor (*Hspa1a* and *Sox9*) markers (Chevalier et al., 2009; Ide et al., 2021; Xu et al., 2025) was determined in all (Figure A8a) or in *Epo* mRNA‐positive (Figure A8b) Visium spots. Although there was a strong general increase in most of these markers in the ligated compared with the contralateral kidney (Figure A8a), none of these markers exceeded the average levels over all Visium spots in the *Epo* mRNA‐positive spots of both the contralateral and ligated kidneys, except the *Havcr1* mRNA, template for the kidney injury molecule 1 (KIM1) (Han et al., 2002; Karmakova et al., 2021), which was strongly increased (Figures 5a and A8b). Of note, the *Acta2* mRNA, template for α‐smooth muscle actin (αSMA), remained one of the lowest members of this panel in the *Epo* mRNA‐positive spots (Figure 5a), arguing against a pronounced (myo)fibrotic transdifferentiation of reactivated REP and/or directly neighbouring cells. Essentially identical data were obtained with kidneys derived from a second mouse treated with roxadustat after 7 days of UUO, with an even stronger increase in *Havcr1* (KIM1) mRNA (levels clearly above average and similar to *Epo* mRNA) and no induction of *Acta2* (αSMA) mRNA in *Epo* mRNA‐positive spots (Figure A9).

Focusing on the remaining Visium spots with the highest proportion of PT S3 cells (following cell type deconvolution), *Acta2* and *Havcr1* were strongly expressed in the ligated kidney (Figure 5b). Focusing on the still abundant *Slc5a1* (Sglt1)‐positive spots, *Acta2* and especially *Havcr1* were highly induced in the ligated kidney (Figure 5c). Several other injury markers (*Lcn2*, *Tgfb1* and *Vcam1*) were also clearly induced in *Epo* mRNA‐positive Visium spots of the ligated compared with the contralateral kidneys (Figures A8b and A9b), although this was not evident from the normalized quantification, because their induction was below the general induction over all Visium spots (Figure 5a). *Lcn2*, template for neutrophil gelatinase‐associated lipocalin (NGAL), is known to be a risk marker for CKD progression (Bolignano et al., 2009). *Tgfb1*, template for transforming growth factor β1 (TGF‐β1), was previously reported to induce anti‐inflammatory and mediate profibrotic tissue remodelling in a dose‐dependent manner (Kayhan et al., 2023). *Vcam1* mRNA, template for vascular cell adhesion molecule 1 (VCAM1), has been suggested as a marker for a failed repair process of the PT (Ide et al., 2021; Kirita et al., 2020).

Sequential immunofluorescence of the marker proteins αSMA and KIM1 confirmed their strong general increase in the ligated kidneys (Figures 5d, e; Table S4), consistent with global (myo)fibrotic tissue remodelling and PT epithelial cell injury, respectively. *Epo* mRNA proximity analyses, as described above, demonstrated that roxadustat activated REP cells proximal to αSMA in addition to KIM1‐positive cells (Figure 5f; Table S4), whilst the REP cells themselves did not show increased αSMA or KIM1 proteins (Figure 5d).

Together, these results demonstrated that myofibroblast (trans)differentiation did occur in tubular cells of the direct REP cell vicinity, but to a lower degree than in other regions of the injured kidney and not in the REP cells themselves. Although Sglt1 levels remained surprisingly high, all PT segments declined, and the remaining PT S3 cells were highly positive for injury markers, including those in the direct vicinity of reinduced REP cells.

### 3.5 Gene ontology and cell‐to‐cell communication analyses of the REP cell microenvironment

**FIGURE 6:**
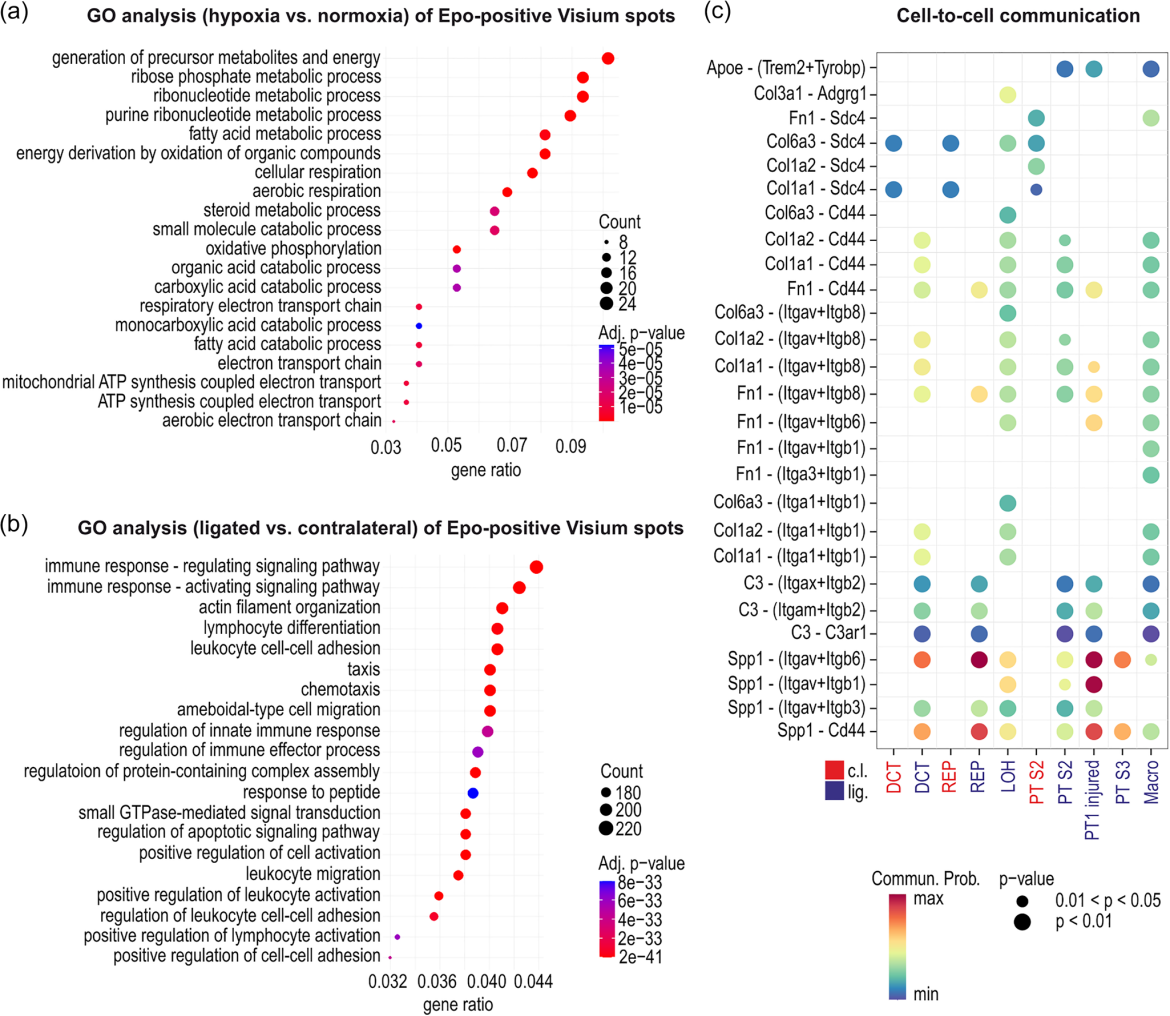
Gene set enrichment and cell‐to‐cell communication analysis in *Epo* mRNA‐positive Visium spots. Gene ontology (GO) analysis in *Epo* mRNA‐positive Visium spots comparing (a) hypoxia (*n* = 2; 2 males) with normoxia (*n* = 2; 1 male, 1 female) or (b) ligated (*n* = 2; 2 males) with contralateral (*n* = 2; 2 males). The 20 most significantly enriched pathways are shown. ‘Count’ refers to the number of differentially expressed genes in each pathway. Enrichment of pathways is indicated by adjusted *P*‐values calculated using hypergeometric tests with Benjamini–Hochberg correction for multiple testing. Gene ratio corresponds to the proportion of input genes that map to a GO term. (c) Predicted ligand–receptor interactions that might contribute to the signalling between the indicated cell types (senders) and *Epo* mRNA‐positive Visium spots (receivers) in kidneys treated with roxadustat following 7 days of unilateral ureteral obstruction. The senders were defined by cell type deconvolution and location within 250 µm of *Epo* mRNA‐positive receiver spots; the cellular nature of the receivers is unknown. Significantly upregulated sender–receiver pairs (if any) in either contralateral (c.l.) or ligated (lig.) samples (*n* = 2 each) are indicated by dots whose colour represents the calculated communication probability (Commun. Prob.) inferred from the expression levels of ligands and receptors in the senders and receivers, respectively. The *P*‐values were computed with one‐sided permutation tests. Abbreviations: DCT, distal convoluted tubule; LOH, loop of Henle; Macro, macrophages; PT, proximal tubule; REP, renal Epo‐producing; and S, segment;

**FIGURE A10:**
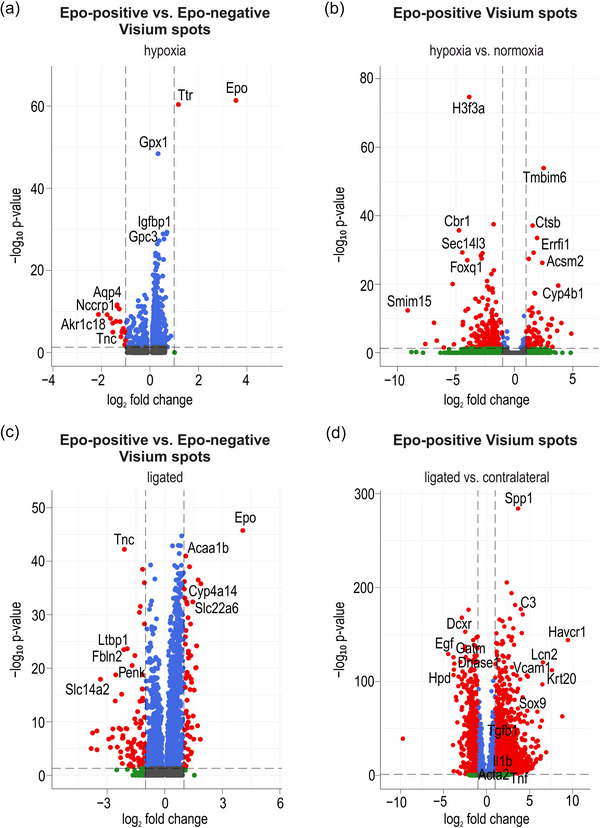
Differential gene expression analyses. Visium mRNA expression data derived from normoxic (*n* = 2; 1 female, 1 male) and hypoxic (*n* = 2; 2 males) kidneys (a, b) and from contralateral (*n* = 2; 2 males) and ligated (*n* = 2; 2 males) kidneys of mice treated with roxadustat following 7 days of unilateral ureteral obstruction (c, d) were analysed. Shown are volcano plots of differentially expressed genes (DEGs) in *Epo* mRNA‐positive versus *Epo* mRNA‐negative spots of hypoxic kidneys (a), hypoxic versus normoxic DEGs in *Epo* mRNA‐positive spots (b), DEGs in Epo mRNA‐positive versus *Epo* mRNA‐negative spots of ligated kidneys (c) and ligated versus contralateral DEGs in *Epo* mRNA‐positive spots (d). Genes on the left or right sides of the dashed lines (red dots) are significantly downregulated or overexpressed, respectively [log2(fold change) ≥ 0.25]. The *P*‐values were calculated from Seurat's implementation of the Wilcoxon rank‐sum test (two‐sided, adjusted for multiple comparisons using Bonferroni correction).

**FIGURE A11:**
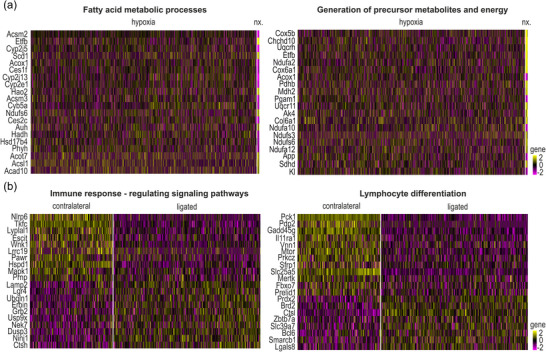
Differential gene expression in specific metabolic pathways. Heatmaps of the indicated exemplary GO‐enriched metabolic pathways within *Epo* mRNA‐positive Visium spots. (a) Metabolic pathways in hypoxic (*n* = 2; 2 males) or normoxic (nx; *n* = 2; 1 male,1 female) conditions. (b) Inflammatory pathways of the contralateral (*n* = 2; 2 males) and ligated (*n* = 2; 2 males) kidneys of mice treated with roxadustat following 7 days of unilateral ureteral obstruction. Colour scales represent *z*‐scores (standardized expression per gene): 0, average *z*‐score of a gene across all samples; +2/−2, *z*‐scores 2SD above or below, respectively, the mean expression of the gene.

Because the metabolic activity of the immediate neighbourhood of REP cells is likely to affect their oxygenation, hence Epo production (Dahl et al., 2022a), we performed pathway enrichment analyses on Visium datasets derived from normoxic and hypoxic kidney samples. As expected, when comparing *Epo* mRNA‐positive with *Epo* mRNA‐negative hypoxic Visium spots, *Epo* mRNA was most strongly expressed (Figure A10a). Differentially expressed genes (DEGs) upregulated by hypoxia in *Epo* mRNA‐positive spots included genes associated with stress responses, such as the anti‐apoptotic and antioxidant *Tmbim6* (Bax inhibitor 1) (Doycheva et al., 2019). In contrast, *H3f3a* (histone H3.3A), which, amongst many other functions, is associated with TGF‐β1‐induced renal fibrosis (Shindo et al., 2018), was strongly downregulated (Figure A10b). Gene ontology (GO) analysis mostly revealed pathways associated with energy metabolic processes that were regulated by hypoxia in *Epo* mRNA‐positive spots (Figure 6a). Heatmaps showing the top 20 DEGs of exemplary GO terms illustrate the downregulation of genes associated with fatty acid metabolism (*Acot7*, *Acsl1* and *Acad10*) and mitochondrial oxidative phosphorylation or closely related components (*Cox5b*, *Cox6a1*, *Ndufa2* and *Chchd10*) in the hypoxic *Epo* mRNA‐positive spots (Figure A11a). These genes are all linked to oxidative catabolism, which is known to be suppressed in hypoxic conditions (Taylor & Scholz, 2022).

When comparing *Epo* mRNA‐positive with *Epo* mRNA‐negative Visium spots of ligated kidneys derived from mice treated with roxadustat following 7 days of UUO, *Epo* mRNA was, unsurprisingly, most strongly expressed (Figure A10c). Apart from the kidney injury markers *Havcr1*, *Lnc2* and *Vcam1* investigated above, DEGs most prominently upregulated by obstruction in *Epo* mRNA‐positive spots included *Spp1* (osteopontin) and *C3* (complement component 3), both of which are known to play key roles in renal fibrosis and CKD progression (Ding et al., 2024; Portilla et al., 2025). In contrast to the hypoxic kidney, GO analysis mostly revealed pathways associated with inflammation and fibrosis (Figure 6b), further confirming the transition from a highly metabolic to an injured REP cell neighbourhood. Heatmaps showing the top 20 DEGs of exemplary GO terms illustrate the differential regulation of genes involved in the immune response in *Epo* mRNA‐positive spots of the ligated compared with the contralateral kidney (Figure A11b).

Cell‐to‐cell communication was investigated by annotating *Epo* mRNA‐positive Visium spots as ‘receivers’ (expressing receptors) and *Epo* mRNA‐negative spots as ‘senders’ (expressing ligands), assessing the most plausible interactions between deconvoluted cell types within a maximal (diffusion) distance of 250 µm (Francis & Palsson, 1997). Amongst the receptors with the highest communication probabilities were the heterodimeric integrin (*Itg*) complexes and the hyaluronic acid receptor (*Cd44*) (Figure 6c). Both are known to serve as *Spp1* (osteopontin) receptors and are involved in cell adhesion and fibrosis progression during CKD (Kim et al., 2010; Matsushita et al., 2024; Shen et al., 2017; Weber et al., 1996). The upregulation of these pathways in the ligated kidneys further confirms the injured REP cell environment.

## 4 DISCUSSION

Since a HIF‐stabilizing agent (FG‐4592/roxadustat) obtained its first global approval for the treatment of anaemia caused by CKD in China in 2018 (Dhillon, 2019; Haase, 2021), the cells capable of Epo production in the kidney have gained new attention. Although the liver might contribute a higher degree to total circulating Epo than in normal conditions, the presence of a non‐functional kidney is still required for sufficient Epo production following treatment with an HIF stabilizer (Bernhardt et al., 2010). Similar results have recently been obtained in mice treated with various HIF‐stabilizing agents (Nakai et al., 2024), leading to the question of what mechanisms are responsible for the loss of Epo production in end‐stage renal disease and the reinduction of Epo by HIF stabilizers.

We previously generated a REP cell fate‐tracking mouse model and reported that roxadustat rapidly reinduced *Epo* gene expression in the damaged kidney in addition to the contralateral healthy kidney (Dahl et al., 2022b; Imeri et al., 2019). Because we found that both repeated hypoxia exposure of healthy mice and roxadustat treatment of UUO mice reinduced Epo to a considerable degree in REP cells that had previously been active, we favoured the hypothesis that acute changes in local tissue $P_{O_{2}}$ not only governed the induction of *Epo* gene expression in the hypoxic kidney but were also responsible for the (reversible) loss of Epo expression in the injured kidney (Dahl et al., 2022a). However, because progression of CKD and tissue remodelling is usually regarded as being associated with tissue hypoxia (Friederich‐Persson et al., 2013; Mimura & Nangaku, 2010; Nangaku, 2006; Tanaka et al., 2014), local tissue hyperoxia as a possible explanation for the loss of Epo production seems to be counterintuitive. Although the lack of Epo induction in the seemingly hypoxic kidney and the finding that less therapeutic Epo is required in high‐altitude residents with end‐stage renal disease (Brookhart et al., 2008) support the ‘local hyperoxia’ hypothesis, alternative explanations for the loss of Epo include inflammatory REP cell‐to‐myofibroblast transdifferentiation (Souma et al., 2013; Suzuki & Yamamoto, 2016), direct inhibition by pro‐inflammatory cytokines (Jelkmann et al., 1992), TGFβ‐receptor 2 signalling‐dependent fibrosis (Fuchs et al., 2021) and healthy residual tissue microregions that are able to compensate for the widespread loss of Epo production following treatment with HIF‐stabilizing agents (Kobayashi et al., 2022).

By using spatial transcriptomics on kidneys derived from healthy hypoxic mice, we showed that the cell types closest to active REP cells, apart from Norn cells themselves, are PT S1–S3 cells on the tubular side and endothelial cells on the interstitial side. Although the endothelial and Norn cell mRNA profiles remained basically the same, pronounced changes were observed in the tubular profile overlapping with *Epo* mRNA‐positive Visium spots of injured kidneys treated with roxadustat following ureteral ligation. In the mouse, UUO is known to cause considerable kidney damage, including progressive tubular loss and tissue fibrosis (Eddy et al., 2012). Increased intratubular pressure and compression of the renal microvasculature create an oxygen‐deprived environment (Fine & Norman, 2008; Kwiatkowska et al., 2023). In these conditions, of all renal cell types, PT cells are the most susceptible to injury, mitochondrial damage and oxidative stress, leading to tubular atrophy and collapse (Forbes et al., 2012). Indeed, cell type deconvolution revealed a shift from PT S1/2/3 to ‘injured’ cells in *Epo* mRNA‐positive Visium spots, confirmed by strongly increased injury and fibrosis markers and corroborated by a switch from metabolic to inflammatory/fibrotic gene set pathways.

After ‘injured PT’ cells, parietal epithelial and LOH cells were the next most highly represented cell types in deconvoluted *Epo* mRNA‐positive Visium spots of injured kidneys. Although this shift might be explained by the massive loss of PT cell‐derived mRNA, it might also imply that a different Norn subpopulation was recruited to another location and cellular neighbourhood. However, for two reasons, it is difficult to draw any conclusions about the exact nature of these potential newly recruited Norn cells. First, as shown in our cell fate‐tracking studies, repeated hypoxia recruits only a fraction of previously tagged cells plus many more previously non‐responding (i.e., non‐tagged) Norn cells (Dahl et al., 2022b). This is in line with our recent report about a severe intrinsic single‐cell heterogeneity even in clonal cell lines and especially of the HIF‐2 dependent hypoxic response (Wilk et al., 2025), suggesting that only a fraction of Norn cells will become REP cells at any given time. Second, the global localization of the reinduced REP cells might change owing to tissue compression, loss and remodelling in the ligated kidneys.

Because sodium transport in the PT exhibits a linear correlation with oxygen consumption (Mandel & Balaban, 1981), the activities of the Sglt2 and Sglt1 cotransporters have a major impact on the oxygen microenvironment (Vallon & Verma, 2021). In normal conditions, Sglt2 is responsible for ∼97% of total glucose reabsorption in the mouse (Rieg et al., 2014; Vallon et al., 2011), suggesting that Sglt2 inhibitors decrease oxygen consumption and increase cortical tissue $P_{O_{2}}$. However, Sglt2 inhibitors have been reported to induce rather than inhibit renal Epo expression (Ghanim et al., 2020; Hare et al., 2021; Marathias et al., 2020; Mazer et al., 2020; Onishi et al., 2020). Although the exact reason for this observation remains enigmatic, Sglt1 might compensate, in part, for the inhibited Sglt2, shifting oxygen consumption further downstream of the PT into the corticomedullary border region (outer stripe of outer medulla). In comparison to the renal cortex, this region is known to have a lower tissue $P_{O_{2}}$ in physiological conditions. Moreover, Sglt2 cotransports glucose with sodium in a 1:1 stoichiometry, whereas Sglt1 is a 1:2 stoichiometry cotransporter, suggesting increased energy demand.

**FIGURE A12:**
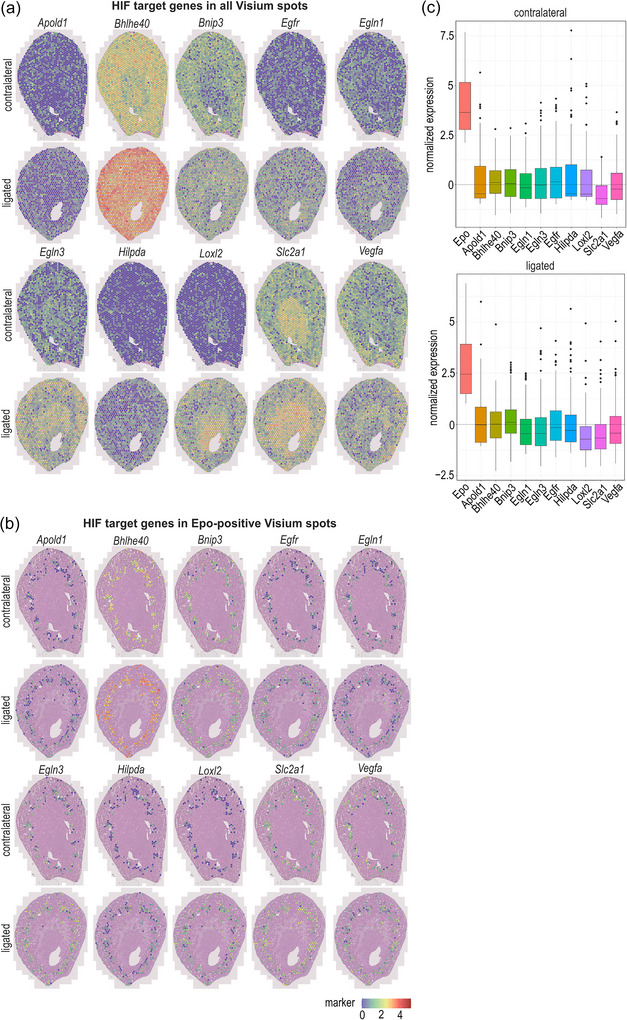
Hypoxia markers in the vicinity of REP cells identified by spatial transcriptomics of an injured mouse kidney. Contralateral and ligated kidneys derived from a mouse treated with roxadustat following 7 days of unilateral ureteral obstruction (same samples as shown in Figure 3). (a, b) Abundance of the indicated HIF target gene‐derived mRNAs in all (a) and *Epo* mRNA‐positive (b) Visium spots. (c) Box plots of the contralateral and ligated kidneys showing standardized Pearson residuals (mean = 0, SD = 1) representing the abundance of *Epo* and the indicated hypoxia marker mRNAs in *Epo* mRNA‐positive Visium spots relative to all Visium spots. A value higher than zero indicates above‐average expression compared with all Visium spots.

To assess biological oxygen availability more directly, it is tempting to analyse HIF‐dependent gene expression in the spatial transcriptomics data. Therefore, we used a panel of hypoxia‐inducible HIF target genes, either known from the hypoxic mouse kidney (*Apold1*, *Bhlhe40* and *Hilpda*) (Kragesteen et al., 2023) or from a list of typically induced genes in hypoxic cell culture (*Bnip3*, *Egfr*, *Egln1*/PHD2, *Egln3*/PHD3, *Loxl2*, *Slc2a1*/Glut‐1 and *Vegfa*) (Wilk et al., 2025). Most of these genes are induced in the ligated kidney when compared with the contralateral kidney (Figure A12a), consistent with the known global tissue hypoxia in injured kidneys. Intriguingly, this induction, if at all, appears to be less prominent in *Epo* mRNA‐positive spots (Figure A12b), especially following quantification (Figure A12c). However, to allow for REP cell identification via Epo gene expression, we analysed UUO mice only after treatment with roxadustat. As a general HIF stabilizer, roxadustat also enhances the expression of all other HIF‐1/2 target genes. Moreover, there are many other factors influencing HIF‐dependent gene expression, including cell type‐specific expression, cellular redistribution, inflammatory modulation and fibrotic remodelling, eventually obscuring HIF target gene expression as a surrogate for oxygen redistribution in the injured kidney. In conclusion, these data should be interpreted with caution and need confirmation by high‐resolution oxygen partial pressure measurements.

Unexpectedly, there was an increased proportion of Sglt1 mRNA (which in the normal kidney is indicative of PT S2/S3 cells) in the *Epo* mRNA‐positive spots of ligated kidneys compared with contralateral roxadustat‐treated UUO kidneys or healthy hypoxic kidneys. Because increased intratubular hydrostatic pressure primarily targets more distal (Sglt1‐positive) segments, the rapid downregulation of Sglt2 might be specific to the injury model and could be attributable to subsequent indirect mechanisms following ureteral ligation. However, considering the pronounced and widespread KIM1 staining, we assume that the strongly Sglt1‐expressing PT cells in the REP cell vicinity are injured and/or dedifferentiated. Although we cannot formally exclude the possibility that such cells are still metabolically active and consume a considerable amount of oxygen, there appeared to be no correlation between Sglt2/Sglt1 and *Epo* mRNA levels, either in the hypoxic kidneys or in the injured kidneys, suggesting that *Epo* gene expression is independent of the degree of glucose transport in its neighbourhood.

## 5 CONCLUSION

**FIGURE 7:**
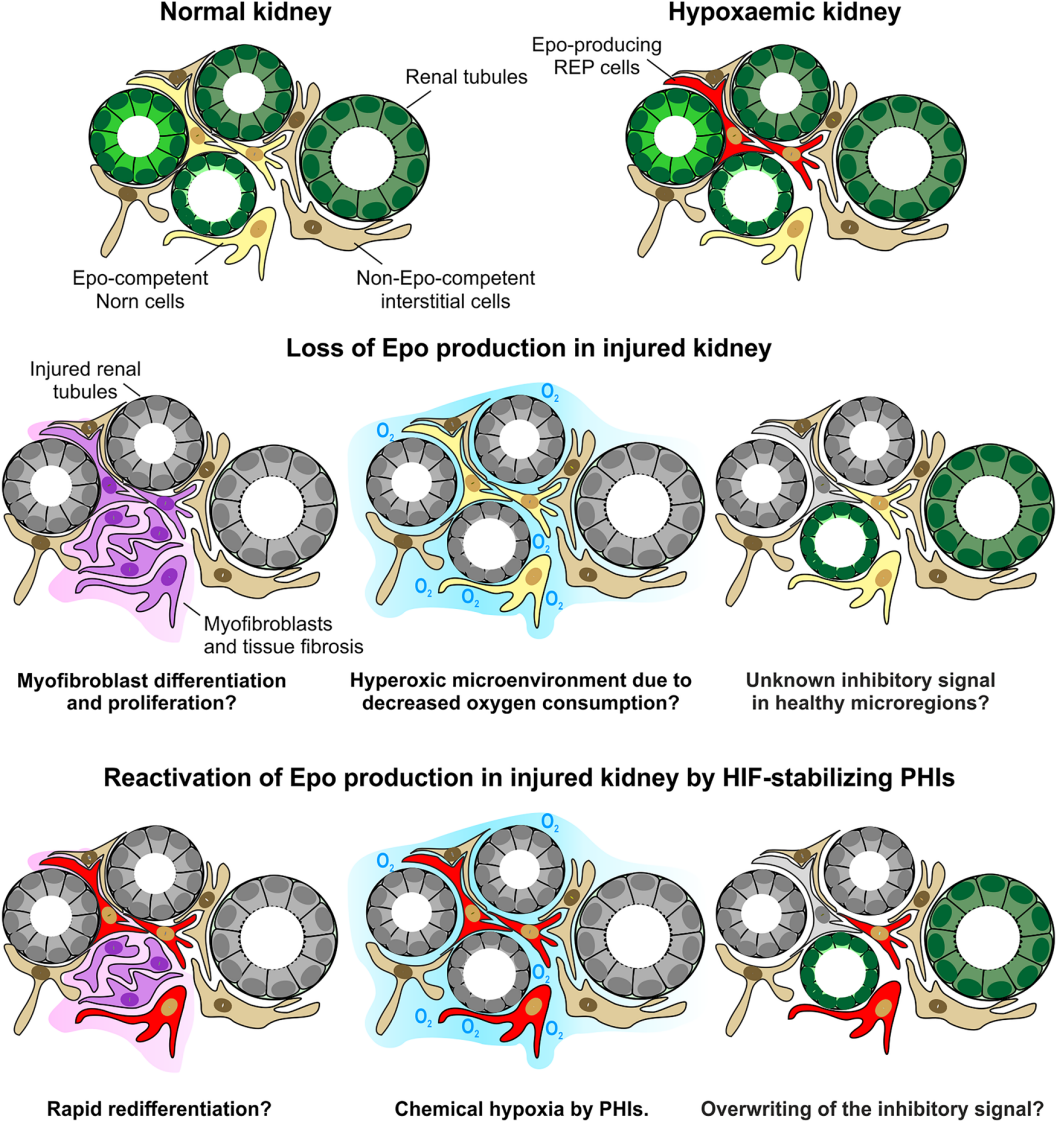
Loss of Epo expression and pharmaceutical reinduction in injured kidneys. Of the three indicated models for the loss of Epo expression in injured kidneys, based on our data, changes in epithelial cell metabolism appear to be the most likely, although not exclusive, explanation. Abbreviations: Epo, erythropoietin; HIF, hypoxia‐inducible factor; PHI, prolyl hydroxylase inhibitor; REP, renal Epo‐producing.

In conclusion, our data support a model according to which, in end‐stage renal disease, Norn cells remain intact in the direct neighbourhood of severely damaged PT. There is no indication for specific differences in myofibroblast transformation, fibrosis, cytokine signalling or tubular epithelial damage in *Epo* mRNA‐containing compared with *Epo* mRNA‐negative Visium spots. Such intact Norn cells represent a prerequisite for the Norn‐to‐REP conversion by HIF stabilizers (Figure 7).

## AUTHOR CONTRIBUTIONS

Olga M. Lempke performed the experiments and analysed the results. Thomas Knöpfel and Stellor Nlandu Khodo provided technical and intellectual input. Roland H. Wenger designed the research and wrote the article. All authors approved the final version of the manuscript and agree to be accountable for all aspects of the work in ensuring that questions related to the accuracy or integrity of any part of the work are appropriately investigated and resolved. All persons designated as authors qualify for authorship, and all those who qualify for authorship are listed.

## CONFLICT OF INTEREST

None declared.

## Supporting information




**Supp. Java Script**. Script to calculate the calibrated distance (in micrometres) from every object of the source class (Epo‐positive cell) to the nearest object of target class (kidney compartment marker).

**Table S1**. Total Visium spot counts, deconvolution‐derived cell‐type spot numbers, and subset of spots selected based on SCT‐normalized *Epo*/*Slc5a1* expression > 0.8 across all kidney slices analysed (data underlying Figures 1–6 and Figures A1–A3 and A5–A12).

**Table S2**. Sglt2/Sglt1 proximity‐based REP cell classes and marker protein signals within 15 µm distance to *Epo* mRNA signals (data of Figure 2c, d).

**Table S3**. Quantification of total Sglt2/1‐positive protein areas and REP cell densities based on the distance between *Epo* mRNA‐FISH and Sglt2 and Sglt1 immunofluorescence signals in contralateral and ligated kidneys (data of Figure 4c, d).

**Table S4**. Quantification of total αSMA and KIM1‐positive protein areas and REP cell densities based on the distance between *Epo* mRNA‐FISH and αSMA and KIM1 immunofluorescence signals in contralateral and ligated kidneys (data of Figure 5e, f).

## Data Availability

All materials and underlying raw data can be obtained from the corresponding author upon reasonable request. The spatial transcriptomics data have been deposited in NCBI's Gene Expression Omnibus and are accessible through GEO Series accession number GSE308511. All primary microscopy image data have been deposited in EMBL‐EBI's BioImage Archive and are available via https://www.ebi.ac.uk/biostudies/bioimages/studies/S-BIAD2492.

## Bioinformatical analyses

### Quality control, filtering and clustering of Visium data

Using the SpaceRanger software (10x Genomics), Fastq sequence files were processed and aligned to the mouse reference genome GRCm38, and Visium spots (55 µm in diameter) were mapped to the corresponding Haematoxylin‐ and Eosin‐stained tissue images. Only confidently mapped reads with valid barcodes and unique molecular identifiers (UMIs) were retained to compute the gene‐per‐spot expression matrix. Spots expressing <1000 UMI counts and <500 genes were excluded. Raw expression data were normalized by applying the SCTransform function that uses regularized negative binomial regression as a statistical model, correcting for sequencing depth, variance of gene expression, and technical noise of the dataset. Gene expression is indicated by Pearson residuals that are treated as normalized expression levels (Hafemeister & Satija, 2019). Principal component analysis (PCA) was performed using the RunPCA function (Korsunsky et al., 2019) on the top 2000 most variable genes, selected by using Seurat's FindVariableFeatures with the default variance stabilizing transformation method (Stuart et al., 2019). This variance‐based method identifies genes with the highest dispersion across spots, enhancing biological signals whilst reducing the influence of technical noise. PCA captures the largest variation and important patterns in complex data by transforming multi‐dimensional results into a new, linearly transformed system. The directions in which the data vary the most are called principal components. This allows for finding different cell types, developmental stages or cell states in the tissue. The top 30 PCA dimensions were used for generating K‐nearest neighbour graphs, which group the most similar spots based on their gene expression profiles using Euclidean distance. These graphs were further transformed into a shared nearest neighbour (SNN) graph using the FindNeighbors function (Stuart et al., 2019). Transformation allows for grouping spots that are similar to their neighbours. Clusters of spots were defined by applying the Louvain algorithm to SNN graphs using the FindCluster function (Waltman & van Eck, 2013).

**TABLE A1:** Messenger RNA fluorescence in situ hybridization probes.

RNAscope probe	Accession no.	Target region	Channel
Mm‐Epo	NM_007942.2	39–685	C1/C3
Mm‐Ubc	NM_019639.4	34–860	C3
Mm‐Polr2a	NM_009089.2	2802–3678	C1
bacterial dapB	EF191515	414–862	C1/C2/C3

**TABLE A2:** Antibodies used for immunofluorescence.

Antigen	Type	Reference	Dilution	Company
Sglt1	rabbit polyclonal	30861‐1‐AP	1:500	Proteintech, Manchester, UK
Sglt2	rabbit polyclonal	24654‐1‐AP	1:1000	Proteintech, Manchester, UK
Ncc	rabbit polyclonal	(Sorensen et al., 2013)	1:5000	PinedaAb, Berlin, Germany
Nkcc2	rabbit polyclonal	(Wagner et al., 2008)	1:2000	PinedaAb, Berlin, Germany
αSMA	rabbit polyclonal	ab5694	1:500	Abcam, Cambridge, UK
KIM1	goat polyclonal	AF1817	1:500	R&D Systems, Minneapolis, MN, USA
Secondary goat anti‐rabbit coupled to Alexa Fluor 647		A‐21244	1:1000	Thermo Fisher, Waltham, MA, USA

### scRNAseq data analysis and cell type annotation

Publicly available scRNAseq data of mouse kidney (accession number GSE197266) were downloaded from www.ncbi.nlm.nih.gov/geo. The filtered gene expression matrix was imported into the R environment (v.4.2.2) and processed with the Seurat R package (v.4.3.0.1; Hao et al., 2024). Expression data were normalized by applying the ScaleData function, which transforms gene expression values to have an average of zero and an SD of one (Korsunsky et al., 2019). This step prevents genes with higher expression from dominating the analysis. The top 3000 genes with the highest variance were computed. PCA was performed using the RunPCA function as described above, and the top 20 principal components (PCs) were selected. Because the reference scRNAseq dataset consisted of multiple samples, PC coordinates were corrected for sample batch by applying the Harmony algorithm, using the RunHarmony function implemented in the harmony R package (v.0.1.1; Korsunsky et al., 2019). PCs were adjusted iteratively to align clusters across sequenced samples whilst preserving variation based on sequenced genes. A K‐nearest neighbours graph was computed and refined further into an SNN graph using the FindNeighbors function, taking the top 20 PCA dimensions as input. Clusters were defined by applying the Louvain algorithm to the SNN graph using the FindCluster function. For cell visualization in two dimensions, uniform manifold approximation and projection (UMAP) was computed with the RunUMAP function (Healy & McInnes, 2024), resulting in minimized distances between similar cells and maximized distances between dissimilar cells. Cluster‐specific genes were identified using the FindMarkers function with the option ‘only.pos = TRUE’ (upregulated markers only) and ‘min.pct = 0.1’ (genes detected in ≥10% of the spots); setting cut‐offs of the false‐discovery rate < 0.01 and log2(fold change) > 1 (Stuart et al., 2019). Clusters were annotated manually to cell types based on cluster‐specific markers and the expression of known cell type‐specific genes. Cells annotated as ‘proximal tubules’ (PT) were isolated and subjected to a new cluster analysis following the procedure described above, resulting in their annotation into distinct PT subclasses (PT S1, PT segment 1; PT S1/S2, PT segments S1/S2; PT S2, PT segment 2; PT S3, PT segment 3; PT PEC, PT parietal epithelial cells; Injured PT S1, injured PT segment 1; Injured PT S2, injured PT segment 2; Repairing PT, repairing PT; Severe Injured, severely injured PT).

### Visium spot cell type deconvolution

Robust cell type decomposition (RCTD), implemented in the spacexr R package (v.2.2.1; Cable et al., 2022), was used for the cell type deconvolution of all Visium samples using a previously annotated scRNAseq dataset as reference (accession number GSE197266). For each spot, probabilistic cell type proportions and weights were computed using the run.RCTD function (with option doublet_mode = ‘full’), which considers all possible cell types from the reference scRNAseq dataset (Cable et al., 2022). Percentages of cell type composition were computed for each spot by normalizing RCTD weights with the normalize_weights function, which converts weights into relative proportions. RCTD weights represent the estimated contribution of a specific cell type to the gene expression profile in a spot (Cable et al., 2022). For each spot, the sum of the weights across all cell types was calculated and divided by the total sum, resulting in a percentage for each cell type. The Pearson correlation between cell type percentages across all spots was estimated, and a cell type interaction graph based on spatial co‐localization within the same spots was computed using the get_spatial_interaction_graph function from the SPOTlight4 R package (v.0.1.7; Elosua‐Bayes et al., 2021).

### ‘Positive spots’

We defined a given Visium spot as ‘positive’ (such as for *Epo* or *Slc5a1*) if the corresponding SCT‐normalized and log‐transformed mRNA counts (stored in the ‘data’ matrix) were >0.8, which corresponds roughly to one UMI count. We compared cell type proportions for PT subsets within *Epo* mRNA‐positive spots between samples from different conditions, applying a Mann–Whitney *U*‐test.

### Interactive box plot tool (shinyapp)

To compare directly the expression of each chosen nephron segment marker in each sample separately, we created an online accessible tool generating box plot graphs, which shows gene expression solely in *Epo* mRNA‐positive Visium spots (https://geneviagb.shinyapps.io/interactive_boxplot_9Lk412mdN/). The list of genes available in the app consists of all genes captured by the probes used in Visium experiments. Box plots represent standardized, variance‐stabilized expression values (Pearson residuals), obtained by applying the aforementioned SCTransform function. For visualization, expression values for selected genes are shown only within *Epo* mRNA‐positive spots. Pearson residuals were further centred and scaled (mean = 0, SD = 1), allowing for interpretation relative to the whole sample. The lower and upper boundaries of the box correspond to the first and third quartiles (the 25th and 75th percentiles), whilst the line inside the box represents the median. For selected genes, a median less than zero means lower than average expression compared with the average expression across all Visium spots in the sample. The upper whisker extends from the upper bound of the box to the highest value within 1.5 × interquartile range, where the interquartile range is the distance between the first and third quartiles. The lower whisker extends from the lower bound of the box to the lowest value within 1.5 × interquartile range. Data points beyond the whiskers are considered outliers and are plotted individually.

### Integration of samples and clustering

To visualize normoxic and hypoxic samples with *Epo* gene expression, we generated a gene‐per‐spot expression matrix merging gene expression vectors of selected samples. Raw expression data were normalized using the SCTransform function, and PCA was performed using the RunPCA function, as described above. To correct for sample batch effects, integration anchors were computed across samples using reciprocal PCA, projecting each dataset into the PCA space of the others to identify mutual anchors for dataset alignment. Anchors were computed with the FindIntegrationAnchors function, with the reduction option set to ‘rpca’ and k.anchor = 20 (Stuart et al., 2019). Each anchor represents a pair of spots that have similar gene expression profiles across datasets, helping to align them properly. Spot integration across different samples was performed by applying the IntegrateData function with option k.weight = 80 (Hao et al., 2021), using the previously computed set of anchors. This produced a corrected gene‐per‐spot expression matrix. On this expression matrix, we computed PCA followed by κ‐nearest neighbours (kNN) graph construction and shared nearest neighbours (SNN) graph computation. Finally, clusters were computed with the FindClusters function at different resolutions (Waltman & van Eck, 2013). Cluster‐specific genes were identified using the FindMarkers function, with option ‘only.pos = TRUE’; assay = ‘SCT’ and min.pct = 0.1; setting cut‐offs of false‐discovery rate < 0.01 and log2(fold change) > 0.8). Clusters were annotated manually to cell types based on cluster‐specific markers.

### Marker gene identification

For the identification of marker genes specifically enriched in *Epo* mRNA‐positive Visium spots, samples were pre‐processed and integrated as described in the previous section (‘Integration of samples and clustering’) and annotated based on the main cell type extracted from the deconvolution. *Epo* mRNA‐positive spots were defined as above (SCT normalized expression > 0.8). Briefly, all samples were integrated using the RCPA method in Seurat (v.5.2.0; Hao et al., 2024). Dimensionality was reduced via PCA performed on the 2000 most variable genes (nPC = 30), and the resulting UMAP plots were inspected to ensure proper integration of all tissues. Marker genes of the *Epo* mRNA‐positive spots were identified using the FindMarkers function from Seurat, based on a Wilcoxon rank‐sum test of Epo mRNA‐positive spots against all others in normoxic, hypoxic, ligated FG‐4592‐treated and contralateral FG‐4592‐treated conditions; on the SCT assay and setting logfc.threshold to 0.25, and min.pct to 0.1. Only genes with Bonferroni‐adjusted *P*‐values of <0.01 and absolute log2(fold change) > 0.25 were considered.

### Differential expression and pathway enrichment analyses

For the evaluation of differences in the transcriptomic profiles of Epo mRNA‐positive spots between samples, we used the SCT assay and applied the MAST framework within Seurat's FindMarkers function to compare hypoxic versus normoxic and ligated versus contralateral FG‐4592‐treated mouse kidney samples (Finak et al., 2015; Stuart et al., 2019). Genes were considered differentially expressed if they had a false‐discovery rate‐adjusted *P*‐value of <0.01 for injured versus healthy, <0.05 for treated versus untreated, and an absolute log2 > 0.25. To explore the biological significance of the differentially expressed gene (DEG) lists, functional enrichment analysis was performed using the clusterProfiler package (v.4.9.0.2) and the org.Mm.eg.db database (v.3.16) (Carlson & Falcon, 2019; Yu et al., 2012). Biological Process Gene Ontology (GO) enrichment pathway analysis was performed using the enrichGO function (Yu et al., 2012). The input lists were ranked by log2, and enrichment results were filtered with a *P*‐value cut‐off of 0.01 and a *q*‐value cut‐off of 0.05. GO term enrichment was assessed using a hypergeometric test with Benjamini–Hochberg correction for multiple testing (clusterProfiler enrichGO; Yu et al., 2012). Heatmaps were created based on the top 20 DEGs for each GO term. The mRNA level of each gene was mean‐centred and divided by its SD, resulting in a *z*‐score that represents relative expression changes per gene.

### Cell‐to‐cell communication analysis

To infer intercellular communication networks in spatial data, we used the CellChat package (v.2.1.2; Jin et al., 2025). The CellChat objects for each sample individually were built using SCT‐normalized data and associated metadata (cell type annotations based on the majoritarian cell type and *Epo* mRNA expression as described above). The analysis focused on identifying ligand–receptor interactions between spatially proximal cell types (interaction.range = 250 µm, scale.distance = 0.01, contact.range = 100 µm, CellChatDB.mouse database). Interaction probability was computed using the computeCommunProb function (Jin et al., 2021), keeping communications with a significant interaction strength (*P* < 0.05) assessed by a permutation test. Network centrality analysis was performed to identify dominant senders and receivers, and interaction patterns were visualized using heatmaps. CellChat analysis was also run on each group of samples (normoxia, hypoxia, contralateral and ligated) and merged into different CellChat objects to evaluate differential communication networks. We calculated the difference in interaction strength matrices between conditions, identifying altered signalling interactions. Differential communication analysis was performed by calculating the difference in interaction strength matrices between conditions, and differential expression of signalling genes between conditions was assessed with netMappingDEG to quantify gene‐level changes within the communication network, filtering genes with an absolute ligand log2(fold change) > 0.25 and *P*‐value of <0.05 (Jin et al., 2021).
