# Supplementary material for: Cellular microenvironment of erythropoietin‐producing cells in hypoxic and injured mouse kidneys
Source: Exp Physiol. 2026 Jan 8;111(4):2236–67. doi: 10.1113/EP093422 (PMC13140485; doi:10.1113/EP093422)
Supplement: Supplementary file 1 — Supp. Java Script. Script to calculate the calibrated distance (in micrometres) from every object of the source class (Epo‐positive cell) to the nearest object of target class (kidney compartment marker). [file EPH-111-2236-s004.pdf]

SUPPLEMENTARY JAVA SCRIPT

This script calculates the calibrated distance (in μm) from every object of the source class (Epo-positive cell) to the nearest object of target class (kidney compartment marker). It converts pixel distances to microns and merges all the annotations of each target class into a single combined multi-object shape to simplify subsequent distance calculations. The shortest distance of an Epo-positive cell to the target class object is calculated as the shortest Euclidean distance from the edge of the source object to the nearest edge point on the target class merged object.

```
// Modified script to calculate distances from one specific class to three specified target classes
// User specifies sourceClass, targetClass1, targetClass2, and targetClass3

print "Caution, this may take some time for large numbers of objects."
print "If the time is excessive for your project, consider size thresholding some objects."

// Define the classes
String sourceClass = "Epo+" // Source class
String targetClass1 = "Sglt1+" // First target class
String targetClass2 = "Sglt2+" // Second target class
String targetClass3 = "Epo+" // Third target class (same as source to find nearest Epo+ cell)

// Get all objects
objectsToCheck = getAllObjects().findAll { it.isDetection() || it.isAnnotation() }

def cal = getCurrentServer().getPixelCalibration()
if (cal.pixelWidth != cal.pixelHeight) {
    println "Pixel width != pixel height ($cal.pixelWidth vs. $cal.pixelHeight)"
    println "Distance measurements will be calibrated using the average of these"
}

// Combine geometries for target classes except for Epo+
Map targetClassObjects = [:]
[targetClass1, targetClass2].each { c ->
    def geom = null
    def classObjects = getAllObjects().findAll { it.getPathClass() != null && it.getPathClass().getName()
== c }
    if (!classObjects.isEmpty()) {
        classObjects.eachWithIndex { o, i ->
            if (i == 0) { geom = o.getROI().getGeometry() } else { geom =
geom.union(o.getROI().getGeometry()) }
        }
    }
    targetClassObjects[c] = geom
}

// Iterate over source class objects and calculate distances
objectsToCheck.findAll { it.getPathClass() != null && it.getPathClass().getName() == sourceClass
}.each { sourceObject ->
    def sourceGeom = sourceObject.getROI().getGeometry()

    // Calculate distances to other Epo+ objects
    double minDistanceEpo = Double.MAX_VALUE
    objectsToCheck.findAll { it.getPathClass() != null && it.getPathClass().getName() == targetClass3
&& it != sourceObject }.each { targetObject ->
        def targetGeom = targetObject.getROI().getGeometry()
        double distancePixels = sourceGeom.distance(targetGeom)
        if (distancePixels < minDistanceEpo) minDistanceEpo = distancePixels
    }
    if (minDistanceEpo != Double.MAX_VALUE) {
        double distanceCalibrated = minDistanceEpo * cal.getAveragedPixelSize()
```

```
        sourceObject.getMeasurementList().putMeasurement("Distance in um to nearest Epo+ annotation", distanceCalibrated)
    }

    // Calculate distances to other target classes
    targetClassObjects.each { targetClass, targetGeom ->
        if (targetGeom != null) {
            double distancePixels = sourceGeom.distance(targetGeom)
            double distanceCalibrated = distancePixels * cal.getAveragedPixelSize()
            sourceObject.getMeasurementList().putMeasurement("Distance in um to nearest " + targetClass + " annotation", distanceCalibrated)
        }
    }
}

print "Done! Distances calculated for source class to specified target classes."
```
